# Magnetic Nanoparticles Used in Oncology

**DOI:** 10.3390/ma14205948

**Published:** 2021-10-10

**Authors:** Veronica Manescu (Paltanea), Gheorghe Paltanea, Iulian Antoniac, Marius Vasilescu

**Affiliations:** 1 Faculty of Material Science and Engineering, University Politehnica of Bucharest, 313 Splaiul Independentei, District 6, RO-060042 Bucharest, Romania; veronica.paltanea@upb.ro (V.M.); antoniac.iulian@gmail.com (I.A.); marius.vasilescu@upb.ro (M.V.); 2 Faculty of Electrical Engineering, University Politehnica of Bucharest, 313 Splaiul Independentei, District 6, RO-060042 Bucharest, Romania; 3 Academy of Romania Scientist, 54 Splaiul Independentei, RO-050094 Bucharest, Romania

**Keywords:** magnetic nanoparticles, cancer therapy, drug delivery, radiosensitizing properties, glioblastoma, magnetic hyperthermia, ferroptosis

## Abstract

Recently, magnetic nanoparticles (MNPs) have more and more often been used in experimental studies on cancer treatments, which have become one of the biggest challenges in medical research. The main goal of this research is to treat and to cure advanced or metastatic cancer with minimal side effects through nanotechnology. Drug delivery approaches take into account the fact that MNPs can be bonded to chemotherapeutical drugs, nucleic acids, synthetized antibodies or radionuclide substances. MNPs can be guided, and different treatment therapies can be applied, under the influence of an external magnetic field. This paper reviews the main MNPs’ synthesis methods, functionalization with different materials and highlight the applications in cancer therapy. In this review, we describe cancer cell monitorization based on different types of magnetic nanoparticles, chemotherapy, immunotherapy, magnetic hyperthermia, gene therapy and ferroptosis. Examples of applied treatments on murine models or humans are analyzed, and glioblastoma cancer therapy is detailed in the review. MNPs have an important contribution to diagnostics, investigation, and therapy in the so called theranostics domain. The main conclusion of this paper is that MNPs are very useful in different cancer therapies, with limited side effects, and they can increase the life expectancy of patients with cancer drug resistance.

## 1. Introduction

Magnetic nanoparticles (MNPs) have been used in nanomedicine and the manufacturing of nanosensors for a long time. It is well known that MNPs have a controlled interaction with an external magnetic field, generated by using permanent magnets; therefore, as a consequence, the position of an MNP could be used to exactly establish where a medical problem exists. An alternating magnetic field induces a thermal heating process in the MNPs, and they are very useful as a contrast substance in nuclear magnetic resonance imaging (MRI) and as drug delivery vehicle for different diseases. The alloys of FeCo, magnetite (Fe_3_O_4_) and maghemite (γ—Fe_2_O_3_) are the most used materials, because a high value of the spin magnetic moment is present [1]. In the case of Fe_3_O_4_ the iron and the oxygen atoms are arranged in an inversed spinel structure, and the γ—Fe_2_O_3_ is characterized by the fact that the iron is bivalent. The bivalent iron could catalyze the oxidation of the hydrogen peroxide, resulting in toxicity due to the reactive species of oxygen. Usually, maghemite particles must have an outer shell, made with biocompatible materials such as gold or other polymers or resins, to prevent the toxicity of this alloy [2,3].

This paper focuses on magnetic nanoparticles used in oncology, and it provides a comprehensive review on this topic, taking into account specific aspects of the neoplasm treatment. The preparation methods of the MNPs used in medicine, classified as chemical, physical, and biological technologies, are presented.

Magnetic nanoparticles’ functionalization is a very important topic, and it will also be taken into account in the discussion. The synthesis process of the MNPs with gold (Au@MNPs) implies two important steps: the synthesis of the iron oxide and the application of a gold shell. The gold shell can be directly applied onto the magnetic core [4,5,6,7] or indirectly, by adding a glue-like material layer between the iron oxide and gold shell [8]. Other inorganic and organic compounds such as silica, silver and polymers are also a viable candidate for MNP functionalization [9]. Today, superparamagnetic nanoparticles of iron oxide with a biocompatible shell (SPIONs) are being used more and more often in personalized medicine for the diagnosis and treatment of different types of cancers [2,10,11,12,13]. With the help of SPIONs, a real-time monitorization of the treatment is possible, by combining SPIONs with different medicines and then by following the changes that appear in the T1 or T2 relaxivity parameters [14]. The multifunctional SPIONs are a very important factor in immunotherapy, because through SPIONs the modulation of the macrophage polarization is possible, and in the case of a human monocyte cellular line, they can be transformed from the M_2_ state (pro-tumorigenic) to the M_1_ state (anti-tumorigenic). As an intermediate effect, antigen transport through lymph nodes is possible and activates the dendritic cells that can cause, as a final result, an important response from the T cells [15].

In the case of “in vivo” application, the hydrodynamic dimension of magnetic nanoparticles becomes one of the most important parameters, being restricted by the anatomical characteristics of the human body and by the fact that this type of materials is delivered in an aqueous solution. Nanoparticles smaller than 150 nm could pass the endothelial barrier, but the blood–brain barrier is permeable for magnetic nanoparticles with reduced dimensions and electric neutral lipidic molecules with a concentration lower than 500 g/mol [16,17,18]. The area to volume ratio is a limitative factor, which is taken into consideration in the nanoparticle synthesis process.

Magnetic nanoparticles tend to form aggregates that are very important if the material is used as a contrast substance in magnetic resonance investigations [19,20,21]. MNPs also possess numerous advantages, such as a large surface area to volume ratio, pore sizes, functionalized surfaces, multiple sites for interaction or binding, and low mass transfer resistance, which makes them very suitable to be used in different disease treatments.

**Figure 1:**
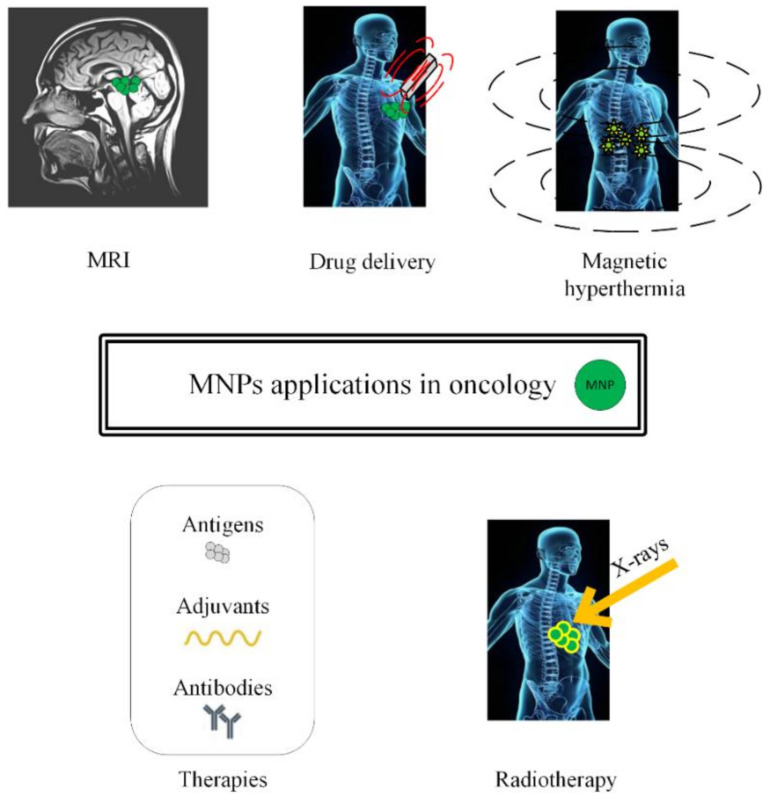
Schematic representation of magnetic nanoparticles’ applications in oncology (MRI investigations, drug delivery in chemotherapy, magnetic hyperthermia, immunotherapy and radiotherapy).

In this review, we will present different approaches to treat cancer by combining chemotherapy, radiotherapy, immunotherapy, and gene therapy with MNPs or SPIONs, as depicted in Figure 1.

Magnetic-induced hyperthermia uses SPIONs to destroy the malignant tissues, which are difficult to treat through conventional therapies, and it will be detailed in Section 5. The tumoral tissues are targeted with SPIONs, whose magnetic properties are enhanced by using an external alternating magnetic field that induces a heating process in the neoplasm area. It is well known that by increasing the tissue temperature above 42 °C, the structural and functional proteins alter and the necrosis of the tumoral tissue begins. 

The ferroptosis process will also be discussed and the side effects of the MNPs or SPIONs treatment will be highlighted in the final section of this review. The challenges of these innovative treatments will be presented.

Today, these treatments are successfully applied in murine animal models, but these approaches are considered very useful in the case of cerebral cancers such as glioblastoma, and they could be lifesaving in patient cases, where classical treatments provide poor results.

## 2. Magnetic Nanoparticles’ Synthesis

In the case of biomedical applications, there are some important restrictions, which must be taken into account. Each medical investigation and treatment requires special and dedicated nanoparticles. The surface effects, the crystallinity grade, and the link between the magnetic properties and the dimensions or shapes of the nanoparticles should be considered. Currently, the ideal shape of a magnetic nanoparticle is spherical or quasi-spherical.

The synthesis process can be divided into three principal categories as a function of the aggregation state of the precursors (gaseous, aqueous, or solid). Another classification can be performed as a function of the process type (chemical, physical, or biological). The most important synthesis methods presented in the literature are co-precipitation, sol-gel procedure, and hydrothermal synthesis [22]. Additionally, new methods were developed, such as microemulsion, thermal decomposition, sonolysis, and biosynthesis [23]. In Table 1, the main synthesis procedures that are used in practice are presented.

**Figure 2:**
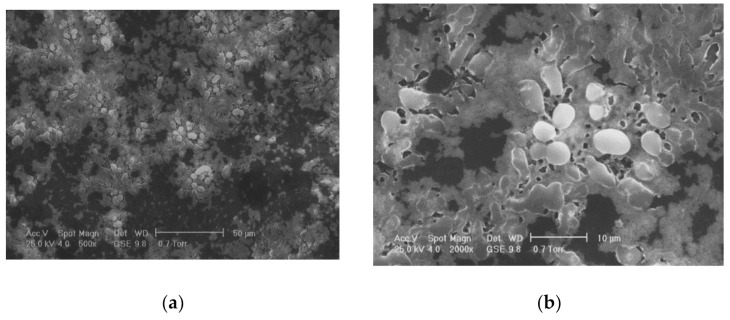

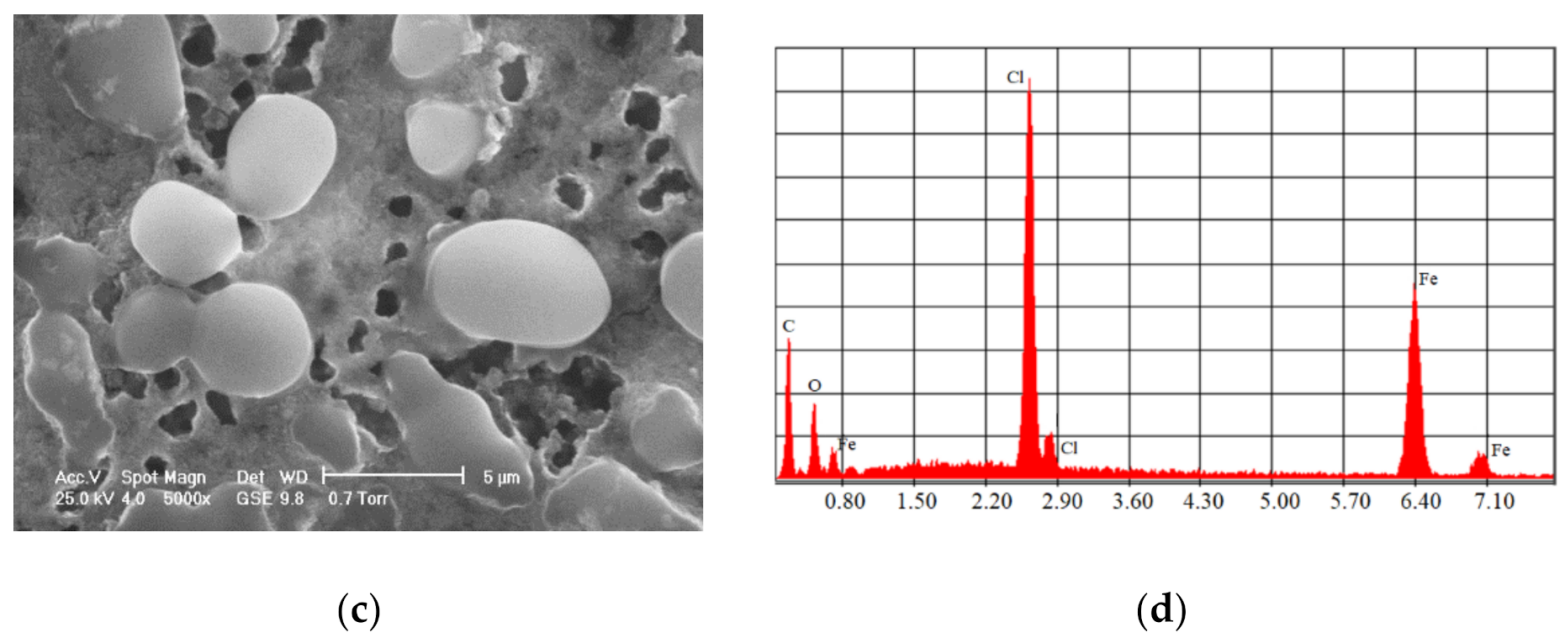
SEM analysis made in the case of Fe_3_O_4_ MNPs obtained through the co-precipitation method at three different magnification scales: (**a**) 500×; (**b**) 200×; (**c**) 5000×; and the resulting elemental analysis (**d**).

One of the most commonly used chemical methods is co-precipitation. The synthesis process of the nanoparticles used in this method can be completed in an open or closed environment. The open medium includes the following steps: co-precipitation, hot organic solvent use, a gaseous route or solid chemical reactions, and the closed medium involves oil- or water-based microemulsions and porous inorganic or organic templates. In Figure 2 are presented scanning electron microscopy (SEM) images of MNPs obtained through the co-precipitation method at different magnification scales.

The co-precipitation in an aqueous solution consists of using Fe^2+^ and Fe^3+^ precursors, dissolved in an aqueous solution and then mixed with reactive substances, in order to precipitate in the spinelic system. In the case of the Massart method [30], a degassed solution of chlorides based on Fe^2+^ and Fe^3+^ is added to an ammonia solution, washed several times, and then stabilized in basic or acid suspensions. The aqueous medium is kept at a low temperature, and thus the co-precipitation method is considered to be one of the most commonly used technologies [31,32].

Other methods are based on hot organic solvents instead of water. When the dimensions of the particles are between 15 and 20 nm, through the co-precipitation method, using a Fe_2_SO_4_ precursor combined with a weak oxidant, high-quality nanoparticles are obtained [33,34,35,36,37].

In the case of hot organic solvents, MNPs with a high grade of crystallinity and a good monodispersing property are synthetized. The main mechanisms are nucleation and growth, and the coordination ligands are used to impose a good dispersion grade of the magnetic nanoparticles, which have a passivate surface. The particle dimension depends on the boiling temperature of the solvent. This technology consists of mixing the substances at room temperature; then an increase in the temperature is supposed to be applied at a value at which magnetic nanoparticles are rapidly formed. These particles have hydrophobic properties, and they are transferred to an aqueous medium and used in biomedical applications. Some FePt or FePt-Au magnetic nanoparticles are obtained based on crystalline iron oxide, formed from an iron precursor such as Fe(acac)_3_, lauric acid or lauryl ammine [37].

Another important method is the growth of magnetic nanoparticles in limitative conditions (growth under confinement) and the dimensions and shape of the nanoparticles are controlled [38,39]. This closed domain could be considered a matrix that represents a barrier used to control the material nucleus growth. This matrix could be beneficial for obtaining special properties of the magnetic material. Organized surfactants such as microemulsions, defined as thermodynamic stable dispersions, and composed of two non-miscible liquids, are usually employed. The surfactant determines microcavity formation, which represents the closure area. In this way, nucleation, growth and particle agglomeration are avoided. A classical case is the Al_2_O_3_ matrix with cylindrical pores [40].

The sol-gel process is based on the hydrolysis and condensation chemical reactions of metal precursors at low temperatures, resulting in the formation of an inorganic network [41]. The conversion of monomers into a colloidal solution determines the sol, and the gel is produced after the evaporation of the solvent, and by introducing particles in the inorganic network. Through this method, single-phase, fine, dense, and homogenous MNPs can be obtained. Although the sol-gel process is environmentally friendly and it is not an expensive method, it presents with a limited efficiency, and the synthesis process is longer than in the case of the co-precipitation method.

The hydrothermal method consists of MNPs’ generation through a mixture between aqueous bivalent metals in acetate solutions with iron nitrate and a carbon nano template at a pH higher than 7. The final product is washed, centrifuged and annealed at 500 °C, in order to eliminate the carbon compounds. Ethylenediamine or citric acid can be used in the synthesis process [42].

Another important method is based on polyol. The metal ions are reduced in a polyol solution and precipitate as nanoparticles with dimensions lower than 100 nm. [43]. MNPs with small sizes can be obtained on dendrimer platforms or by using ultrasound waves [44]. Electrochemical deposition of metal is used to synthetize MNPs, by reducing the metallic ions dissolved by anode and by depositing them at the cathode. Pyrolysis, Fe^3+^ spray solution or the CO_2_ laser heating process of gases that contain iron precursors were also reported as synthesis methods for MNPs [43].

The biological synthesis technologies are based on plants (bark, leaf, root, fruit, seed, flower), fungus, bacteria, algae, virus, and marine organisms, and they are considered simple, low-cost, non-toxic and eco-friendly. Two examples, applied in the case of iron-based MNPs, are presented in Table 1.

Another preparation method consists of metal–organic material structures, inside which the metallic ions are combined with organic molecules. The inorganic matrices have superior chemical and mechanical properties. The most used inorganic matrix is that based on silica, but carbon nanotubes or materials with a structure similar to graphene or titania nanotubes are also seen in practice. The magnetic nanoparticles are encapsulated in silica, by using ammonia hydroxide through the Stoeber method [45]. Carbon nanotubes were treated with nitric acid in an FeCl_3_ suspension [46].

The gaseous state synthesis is very suitable for the manufacture of magnetic nanoparticles with dimensions lower than 5 nm. This method is based on the formation of gaseous substances oversaturated with vapors due to chemical reactions. The oversaturated vapor state contains atoms or molecules of a material, set at a higher pression than vapor pression. New particles are nucleated through laser pyrolysis or using carbonyl precursors. The pyrolysis is performed using the heating process of a gaseous mixed compound with the help of a continuous laser wave, which initiates and maintains the decomposition reaction. Once an energy level is reached, the metal atoms are produced through a fast coalescence procedure, resulting in nanoparticles which can supplementarily react with another gas components [47,48].

Through solid synthesis, MNPs with sizes lower than 20 nm can be obtained, but this method is rarely used. 

The chemical and physical methods are considered conventional technologies and they are characterized by high costs and complex procedures. Co-precipitation and thermal/hydrothermal decomposition are the most commonly used methods because they are easy and secure.

## 3. Functionalization of Magnetic Nanoparticles

The encapsulation and coating of magnetic nanoparticles represent two important steps, which could be adopted in the MNPs synthesis for medical applications. Usually, hydrophilic substances are used, which are suitable for blood circulation and protein adhesion. Sometimes, thin polymers can be utilized [49,50]. If an organic matrix is involved, the human body does not present an immune response. Synthetic or natural polymer matrices made from polysaccharides or polyesters using the co-precipitation method are also a suitable solution.

The type of the coating which is applied on an MNP is important, because the shell layer could enhance or reduce the magnetic properties of the core, the shell thickness, and the size of the nanoparticles. The main methods used for MNPs’ functionalization are performed in situ or post synthesis, involving mechanisms such as ligand addition, ligand exchange or encapsulation.

### 3.1. Innorganic Compounds

In the case of inorganic compounds, a specific group of ligands should be chosen, in order to manifest a high affinity for MNPs. The inorganic materials used in functionalization process are gold, silica, or silver.

*Gold* is an inert metal, and it is resistive to oxidation and corrosion. Nowadays, there are a high number of techniques associated with gold deposition, such as sputtering, thermal evaporation, and chemical deposition. 

Magnetic nanoparticles for medical applications, functionalized with gold, can be considered as a magnetic core covered with a gold shell (Fe_3_O_4_@Au).

**Figure 3:**
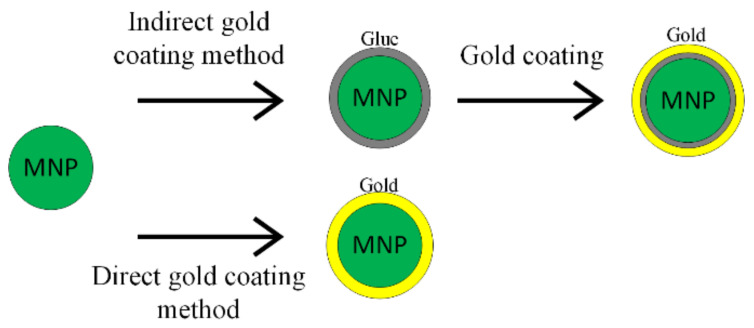
Gold coating methods’ schematic representation.

The synthesis procedure of Fe_3_O_4_@Au nanoparticles includes two steps: firstly, the iron oxide synthesis and secondly the gold shell application. There are two principal methods used to obtain a gold shell, as shown in Figure 3. The first one is called the direct method, where the gold shell is directly formed on the magnetic material; in the second case—the indirect method—a glue-like intermediary material is used [51].

The gold shell can be applied when the magnetite particles are placed in an aqueous solution or in an inorganic material. For the aqueous solution case, the gold shell is created by reducing the Au^3+^ ions through ammonium citrate or borohydride [52]. A disadvantage of this method consists of the fact that if the experimental conditions are not entirely respected and controlled, individual gold nanoparticles appear, and outer gold shell formation becomes impossible. When the magnetic core is included in an organic phase such as oleic acid, the gold outer shell can be formed [53]. The synthesis of Au@MNPs is possible when a non-polar solvent is present, and the obtained gold shell is thick.

Another method in gold functionalization is inverse micelle technology, which is a complicated procedure in which centyltrimethylammonium bromide, octan and 1-butan are used as surfactants and oil is used as a co-surfactant [52].

**Figure 4:**
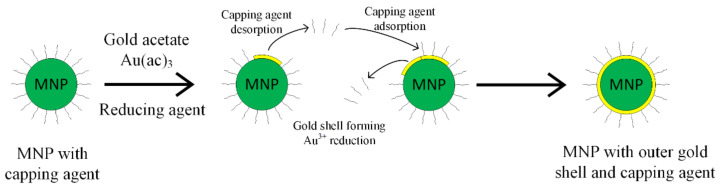
Capping agent desorption and gold coating principle.

The gold shell can also be obtained through the indirect method, interposing a glue layer between the magnetic core and the gold outer shell, as presented in Figure 4 [52,53]. The gold shell must completely cover the magnetic core, in order to ensure a high chemical and physical stability. It has to contain a large number of chemical groups, such as poly-L-histidina, cross-linked polymer (cyclotriphosphazene-co-4, 4′-sulfonyldiphenol) (PZS), polyethylene-imine or silica that can chelate with metallic ions.

When the gold shell was formed on a substrate as silica, the co-precipitation method was applied to synthesize the magnetic core and then sol-gel technology, based on the hydrolysis process of tetraethyl orto-silicate, was used to obtain the silica cover. Three layers of positive charged poli-electrolytes are formed on the magnetic nanoparticles covered with silica. The Au ions are negatively charged, and a gold shell can be obtained through the self-assembly method. Gold shells thicker than 30 nm were reported in [54,55].

The functionalization of MNPs with gold presents several advantages in biomedical applications, because it has a positive impact on the specific sensitivity and compatibility. Au@MNPs can form aggregates due to the magnetic attraction forces developed between particles. The surface changes play an important role in the stability increase, which is affected by the electrostatic exchanges on the material’s surface. The gold shell prevents the metal ion emission from the magnetic core, increasing the MNPs’ biocompatibility [56,57].

*Silver coatings* facilitate the germicidal action of the MNPs, and they are very similar to gold coatings. Silver can generate core–shell structures and heterostructures. Hybrid nanoparticles of Fe_3_O_4_@C@Ag were used for drug release, MRI and two-photon fluorescence imaging, presenting advantages due to having on their surface both carbon and silver.

*Silica* (SiO_2_) is functionalized with MNPs through a similar process to ligand absorption. A silica coating inhibits the aggregation of the MNPs and improves the chemical stability and biocompatibility of the shell–core assembly. Two main methods have been developed to stabilize the silica coating. The first one consists of shielding the magnetic dipole interaction with the silica coating, and the second one is based on charging the MNPs with negative charge, so an electric repulsion phenomenon appears between the shell and the core. SiO_2_@MNPs could be prepared according to the Stoeber method, in which SiO_2_ is generated through hydrolysis and condensation of a sol-gel precursor (tetraethyl orthosilicate, TEOS). As an example, in [58,59], 200 mg of Fe_3_O_4_ was introduced in an ethanol suspension under ultrasonic irradiation for 30 min in nitrogen atmosphere. Then, 11 mL of NH3 (28%) and 400 μL of tetra-ortho-silicate (TEOS) in the Fe_3_O_4_ suspension were sonicated for 2 h. The final compound, Fe_3_O_4_@SiO_2_, was washed in ethanol and then collected through a magnetic separation procedure. 

The second method is the silica deposition from silicic acid solution, and through this method, the particle size can be effectively controlled. Additionally, a higher percentage of the MNPs’ surface can be covered.

The emulsion method consists of controlling the silica shell using micelles or inverse micelles. This procedure is based on the separation of the core–shell MNPs from surfactants linked to the emulsion system.

A main advantage of silica coatings is the presence of surface silanol groups that react with coupling agents, and ligands can be attached to MNPs. In this way, amine groups were introduced on the surface of Fe_3_O_4_@SiO_2_ through hydrolysis and condensation of an organosilane (silanization method). Biomolecules could be easily added by using alkoxysilanes with active groups.

Another method for obtaining potential vectors for drug-delivery treatments involving magnetic nanostructures is based on silica mesoporous structured materials (MCMs). Usually, nonmagnetic or magnetic silica nanoparticles are prepared using the sol-gel procedure based on silica xerogels, hydrothermal synthesis and laser pyrolysis. MCMs present a large surface zone and small pores with dimensions between 15 and 100 Å. The pore openings are hexagonal (MCM-14), cubic (MCM-48) and lamellar (MCM-50) ordered. In [60], the drug miltefosine (hexadecyl 2-(trimethylazaniumyl) ethyl phosphate), used for different fungi, bacteria, cancers and parasite species, was loaded onto a new composite mesoporous silica drug carrier 20–30 nm in size, which contains magnetic nanoparticles modified with NH_2_ and PEG chains. It was concluded that about 11–25% of the drug content was deposited inside the pores of the silica carrier. Miltefosine loaded onto PEGylated magnetic silica magnetite nanoparticles exhibits a very good antiproliferative activity and antineoplastic potential against human tumor HUT-78 cell line (cutaneous T lymphocytes). This material exhibits important properties as a nanocarrier of miltefosine.

In the nanoscale range, nanoporous silica particles can also be used that can cover the MNPs and embed different oncological drugs on the silica support. A SO_3_H-functionalized magnetic nanoporous silica/polymer nanocomposite was developed in [61]. Spherical 100 nm particles were synthesized that had the anticancer drug mitoxantrone (MTX) loaded in the magnetic nanoporous silica support, covered with a chitosan layer infused with prednisolone (PRD). The final coating layer consisted of alginate. The cytotoxic activity was assessed against HD-MY-Z (Hodgkin lymphoma), EJ (urinary bladder carcinoma) and K-562 (myelogenous leukemia) cellular lines, and this innovative material shows great potential in pharmacological applications.

### 3.2. Organic Compounds

**Figure 5:**
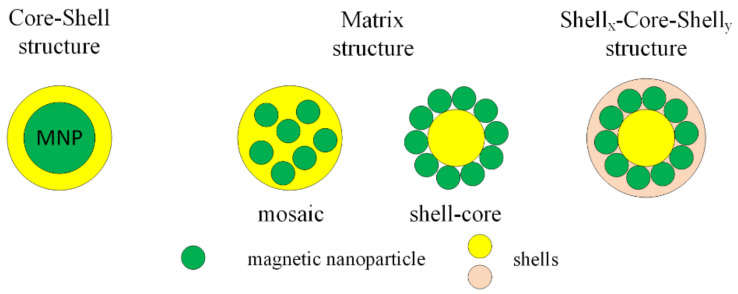
Classification of functionalized structure types with organic compound.

MNPs can be functionalized with organic compounds after or during the synthesis process. Organic functional materials present a good biocompatibility and biodegradability and do not have an important influence on the magnetic properties of MNPs. The functional groups are carboxyl, aldehyde, hydroxyl and amino, which, after the functionalization of MNPs with organic compounds, can be linked to deoxyribonucleic acid (DNA), proteins, enzymes or antibodies. After the functionalization process, the final structure is classified into three types: core–shell, matrix (mosaic or shell-core) and shell_x_–core–shell_y_ (Figure 5).

The most important organic compounds that can be used in the functionalization of MNPs will be detailed and explain in the review.

**Figure 6:**
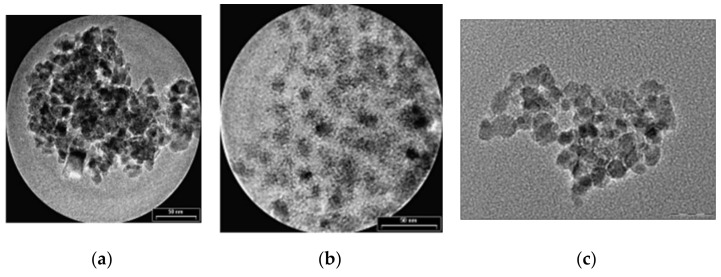
TEM images of Fe_3_O_4_ MNPs: (**a**) unfunctionalized MNPs (average medium size of particles 8.5 ± 2 nm); (**b**) citrate-functionalized MNPs (average medium size of particles 9.5 ± 1 nm); (**c**) phosphate-functionalized MNPs.

The first type of organic compound is the *monomer* class that includes *citrates* and *phosphates*. The surface of MNPs is stabilized in an aqueous solution through the adsorption of citric acid, which have carboxyl groups. The acid is adsorbed on the nanoparticle surface with the help of carboxylate functionalities. This process is dependent on the size and the shape of the particle. MNPs functionalized with citrates are characterized by a negative charge on the particle surface, generating hydrophilic properties. In Figure 6, transmission electron microscopy (TEM) images of Fe_3_O_4_ nanoparticles synthetized through the co-precipitation method are presented. It can be observed that the unfunctionalized MNPs tend to form aggregates, since the citrate-functionalized MNPs present a spherical shape and a relatively uniform dispersion.

Regarding the phosphates group, alkanesulfonic and alkanephosphonic acids can be used as surfactants. After the functionalization with phosphates, the dispersions of MNPs are thermodynamically stable. This method was used to prepare SPIONs, based on magnetite, through co-precipitation of bivalent and trivalent iron ions, using poly(vinylalcohol phosphate) (PVAP). The size of the particles is strongly dependent on the PVAP concentration [50]. From Figure 6c, it can be seen that MNPs functionalized with phosphates tend to form aggregates, a fact that makes them very useful in MRI investigations.

If *small molecules* are employed in MNP functionalization, they are characterized by a low value of the hydrodynamic radius, a fact that is beneficial for nanotechnology. *Short chain aminosilanes* or *amines* are usually used to coat the MNPs’ surface. Magnetite nanoparticles, functionalized with these substances, were considered as being highly water-stable. Another type of small molecule is thiol as an organosulfur compound, 2,3-meso dimercaptosuccinic acid (DMSA), which is characterized through two thiol and two carboxyl groups. It was observed that thiol-functionalized nanoparticles are stable under physiological pH. The stability of the coating could be increased if DMSA is protected through a polyethylene glycol (PEG) chain.

In the *macromolecules* class, natural or synthetic polymers are intensively added for MNPs’ functionalization. A polymer coating balances the van der Waals and magnetic attraction forces, which acts on MNPs. Coating approaches, performed in situ and post-synthesis, are based on reaction mixtures that are simultaneously combined with polymer functionalization. Ligand exchange and ligand adsorption are used together to provide a stable structure. In the case of MNPs’ functionalization with polymers, the agglomeration of the particles is prevented, and different surface properties are obtained.

**Figure 7:**
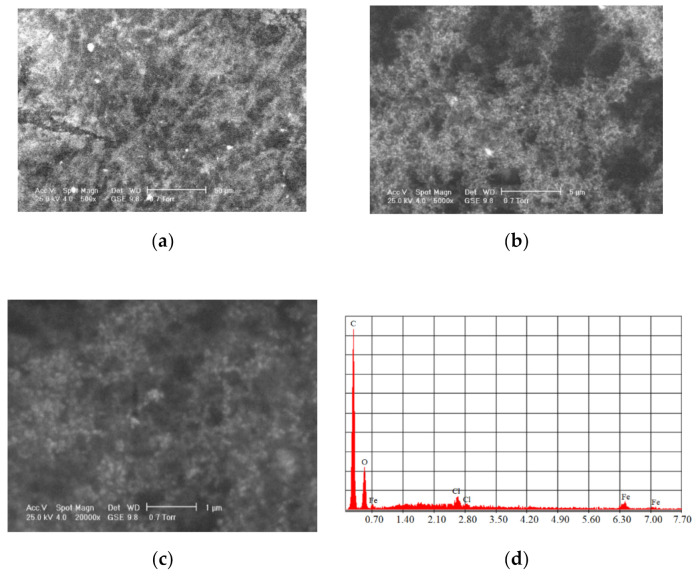
SEM analysis made in the case of Fe_3_O_4_ MNPs obtained through the co-precipitation method functionalized with chitosan at different magnification scales: (**a**) 500×; (**b**) 5000×; (**c**) 20,000×; and the resulting elemental analysis (**d**).

The most commonly used *natural polymers* are *dextran* and *chitosan*. Dextran increases the MNPs’ biocompatibility and is a polysaccharide composed of R-D glucopyranosyl. This polymer’s presence limits the particle size, and it has the advantage that it can be aminated using ammonia, which permits conjugation with complementary chemical groups. Chitosan is alkaline-based, biodegradable, biocompatible, and hydrophilic. MNPs functionalized with chitosan have shown a high MRI contrast and they exhibit very good properties as carriers in different types of treatments [62]. Figure 7 shows SEM analysis performed for Fe_3_O_4_ functionalized with chitosan.

*Polyethylene glycol* (PEG) is a synthetic, hydrophilic, and biocompatible polymer. PEG-coated magnetite nanoparticles present solubility and stability in physiological and aqueous media. Post-synthesis coating technology is preferred in the case of PEG functionalization. *Polyvinil alcohol* (PVA) is a synthetic polymer that can be transformed into gel, and it is highly biocompatible and hydrophilic. A PVA coating prevents self-agglomeration, and the colloidal stability of the PVA-coated particles is good. *Alginate* is another type of synthetic polymer, and it can be characterized as an electrolytic polysaccharide with carboxyl groups, which interact with iron, resulting in the stable assembly of alginate MNPs due to electrostatic repulsion.

**Figure 8:**
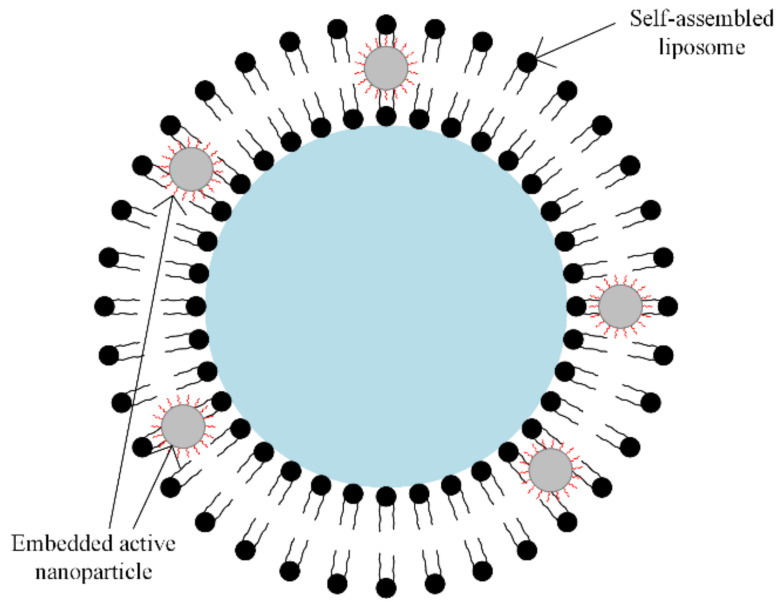
Self-assembled liposome combined with nanoparticles. Solid or water-soluble drugs can be included inside the core region (blue zone) and between interfaces (black spheres); embedded active nanoparticles or oil-soluble drugs in the lipid bilayer can be included.

A special class of synthetic polymers is the *liposomes*. In magnetic drug targeting therapy, the liposomes play the role of carriers, because they present a large encapsulation domain, and biomolecules such as DNA or proteins can be encapsulated by liposomes as one unit [63,64]. The liposomes can be combined with different types of magnetic particles, using the dialysis process of a single unilamellar vesicle or through the extrusion of a combination between phospholipids and magnetic particles, as presented in Figure 8.

*Micelles* or *micellar polymers* can be used as a coating layer for MNPs, and they are made from self-assembled amphiphilic polymers, which contain both hydrophobic and hydrophilic groups. The hydrophobic core helps to facilitate the hydrophobic components’ movements, and the hydrophilic shell determines the MNP’s high stability in the biologic medium [65,66]. The micelles are benefic for MNP aggregation, in the case of contrast substances used in nuclear magnetic resonance [67].

**Figure 9:**
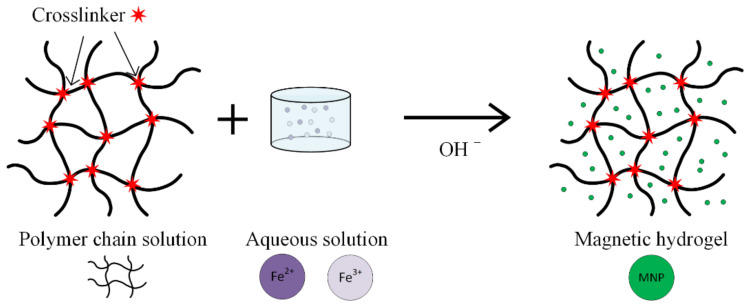
Schematic representation of the in situ co-precipitation method used to prepare a magnetic hydrogel.

*Hydrogels* are considered one of the most promising substances, used in magnetic nanoparticle synthesis for medical applications (Figure 9). These are networks of hydrophilic polymers that contain a high amount of water or physiological fluids. The hydrogels exhibit a high biocompatibility and important therapeutical properties [40,68,69,70,71,72]. When nanogels are used, interactions between materials and living cells are possible, and different types of drugs can be delivered.

Polymeric nanogels in aqueous medium are obtained through the co-polymerization of N-vinylcaprolactam or vinylimidazole in aqueous solution in the presence of a link agent and a reaction initiator, soluble in water [70]. The high grade of porosity of these nanogels permits their use as templates for MNPs, through the hydrolysis reaction of FeCl_2_ or FeCl_3_.

The *epoxy resin* group is useful for different material functionalization processes. SU8 UV photosensitive materials are biocompatible, and they are suitable for the preparation of biochemical coupling structures. The epoxy resin group reacts with nucleophile substances (NH_2_) through a special mechanism. It is also possible to perform the transformation of an epoxy group in a hydroxyl group through a hydrolysis chemical reaction that is based on using an acid, basic or capping agent (i.e., ethanolamine). A primary amine reacts with a carboxylic acid or with an epoxide. The basic characteristics of the solution (with a pH of 9) are suitable for this reaction, and the process is followed by deprotonation [73,74].

The side effects of the hydrolysis reaction can be avoided by using an organic solvent such as dimethylformamide (DMF). Regarding the chemical link agents, there are electrophile or nucleophile substances. The electrophile materials accept electrons, and they are epoxy resins, NHS esters, aldehyde R-CHO and carboxylic acid R-COOH, and the nucleophile materials release electrons (primary amine R-NH_2_, thiol R-SH, alcohol R-OH and water H_2_O) [58,59,75,76].

**Figure 10:**
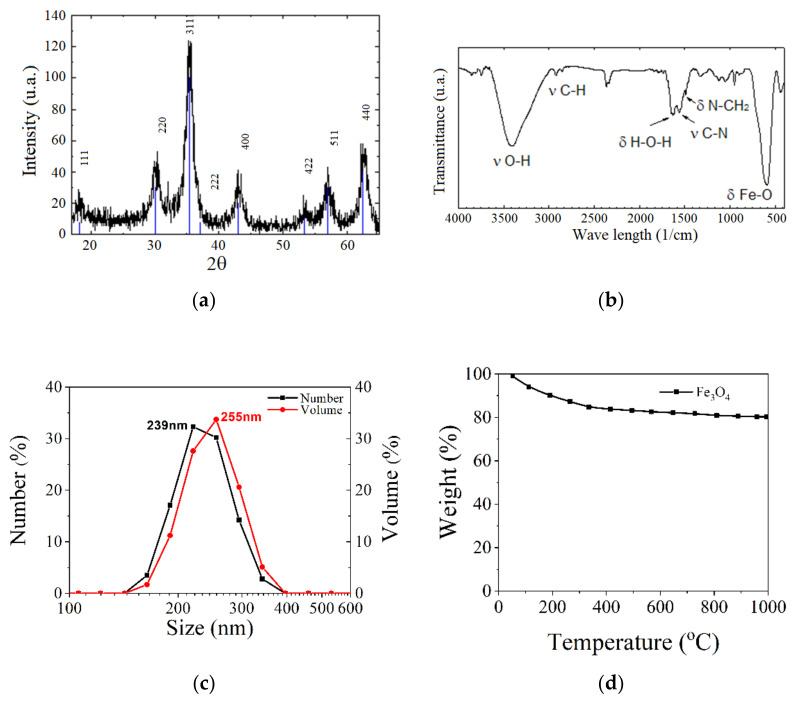
Different types of analysis made on Fe_3_O_4_ nanoparticles: (**a**) X-ray diffraction (XRD); (**b**) FTIR analysis; (**c**) DLS investigation; and (**d**) TGA analysis.

The material morphology could be investigated through infrared spectroscopy, FT-IR transform, X-ray diffraction (XRD), X-ray energy dispersive spectroscopy (EDX), thermogravimetric analysis (TGA), dynamic light scattering (DLS) and zeta potential measurements. The dependence between magnetization and the applied magnetic field is usually measured with a SQUID or vibrating sample (VSM) magnetometers [62]. In Figure 10, different types of analysis made in the case of Fe_3_O_4_ nanoparticles are given as examples.

## 4. Magnetic Properties of MNPs

Usually, MNPs are considered to exhibit a ferrimagnetic, ferromagnetic or superparamagnetic behavior. Below a given size of the nanoparticle, which depends on the material type, MNPs are characterized through a zero value of the magnetic moment, in the absence of an external magnetic field, and they are called superparamagnetic particles (SPIONs). The magnetic moment rapidly increases when a magnetic field is acting on MNPs, and it is oriented along the field direction.

**Figure 11:**
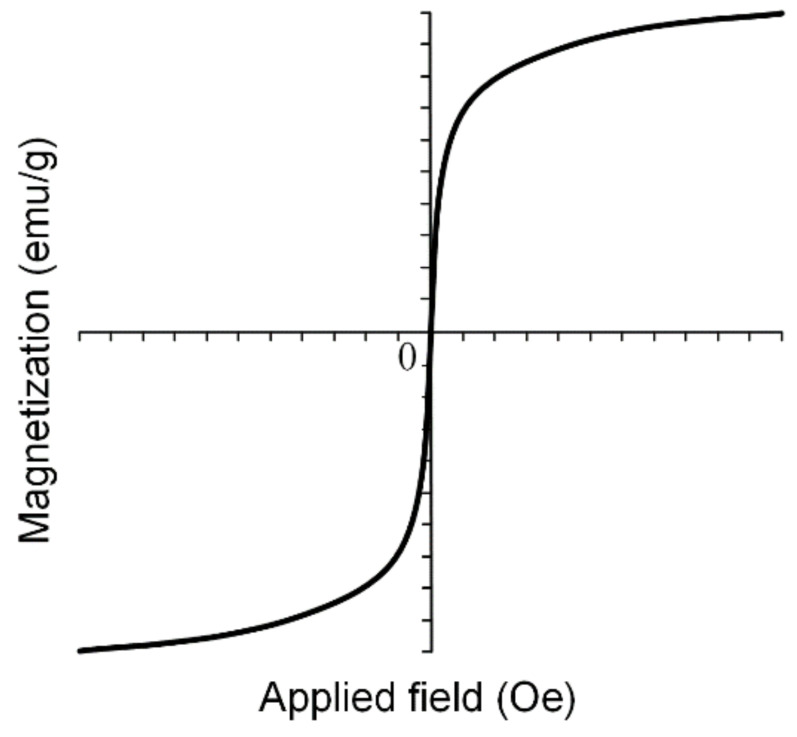
Magnetization curve for SPIONs can be modeled using sigmoidal functions. These types of particles do not present hysteresis phenomena, and so after external magnetic field removal, they do not tend to form aggregates.

SPIONs are very important in medical applications such as magnetic drug targeting, MRI and different cancer treatments due to the fact that when the external magnetic field is removed, SPIONs do not exhibit any magnetic properties and the aggregate formation is prevented. Superparamagnetic nanoparticles present a strong response to external fields, and so they provide a high control of the medical application. In Figure 11, an example of a magnetization curve for iron-based nanoparticles in a superparamagnetic state is shown.

MNPs are characterized through saturation magnetization *M_s_* (maximum induced magnetization), remanent magnetization *M_r_* (the value of the magnetization, which can be measured after the magnetic field is removed) and coercivity *H_c_* (magnetic field strength that is necessary to reduce the magnetization value to zero). In the case of superparamagnetic materials, the dependence *M*(*H*) follows a sigmoidal function, and it presents no hysteresis phenomena [77].

**Figure 12:**
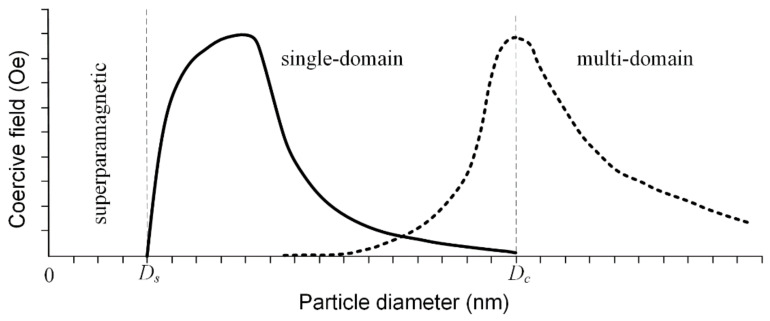
Coercive field variation as a function of the particle diameter, considering the transition between superparamagnetic state to single-domain and to multi-domain state. For the single-domain case, the coercivity variation follows the solid graphical representation in the case of particles that do not interact between them and the dashed line, for the coupled particles.

The saturation magnetization is a function of the temperature, and it is well known that above a so-called blocking temperature, *T_B_* ferrimagnetic and ferromagnetic materials have a superparamagnetic character. This behavior is due to the difference between measuring time and relaxation time as follows: if the relaxation time is lower than the measuring time, the particles are in a superparamagnetic state; otherwise they are in ferrimagnetic or ferromagnetic working conditions [78,79]. When the MNPs’ diameter is kept below a critical value during the synthesis procedure, they are characterized by a single domain structure, and at the lowest diameter value, they are in a superparamagnetic state. If the diameter increases after this critical value, the particle tends to develop a multi-domain magnetic structure (Figure 12). In the case of Fe_3_O_4_ particles, the value of the diameter at which superparamagnetic behavior appears (*D_s_*) is around 25 nm, and the critical diameter for the mono-domain magnetic structure (*D_c_*) is 82 nm. In the case of γ—Fe_2_O_3_, these values are *D_s_* = 30 nm and *D_c_* = 90 nm [80].

The size of the MNPs and the synthesis method are directly linked to the saturation magnetization. The value of saturation magnetization *M_s_* proportionally increases with particle size, and finally it becomes equal to the bulk magnetization value, independent of the synthesis method. Regarding the coercivity field value *H_c_*, it has a similar behavior, but after a specific size value is reached, a transition from a mono-domain to a multi-domain structure occurs, and so, as a consequence, a decrease in coercivity is observed [81,82,83]. In Table 2, the abovementioned magnetic properties in the case of Fe_3_O_4_ nanoparticles are summarized.

The shape of the magnetic nanoparticles is also an important parameter. Refs. [84,85] analyzed the influence of this parameter, and it was concluded that Fe_3_O_4_ spherical nanoparticles have a higher value of T_B_ than cubic and spherical Zn_0.4_Fe_2.6_O_4_ MNPs, and cubic particles exhibit a higher saturation point when compared with other shapes of nanoparticles.

The magnetic properties of MNPs appear due to the presence or absence of unpaired valence electrons, which are placed on the metal atoms or metal ions. Chemical composition is a very important parameter, and it depends on precursor concentration, synthesis method and dopant nature. The solvent could have an influence on the magnetic state, and so in [78], it was observed that when benzyl alcohol was used, Mn-doped ZnO became paramagnetic and Co-doped MNPs exhibited ferromagnetic properties.

The Curie temperature *T_c_* of the MNPs, defined as the temperature above which magnetic nanoparticles have a zero value of magnetization, can be variated through chemical composition. This parameter should be carefully controlled, in order to avoid the overheating phenomenon in magnetic hyperthermia. As an example, in the case of aluminum content variation in Y_3_Fe_5-x_Al_x_O_12_, with x between 0 and 2, the *T_c_* was measured between –40 and 280 °C [78].

## 5. Applications of Magnetic Nanoparticles in Cancer Therapy

The iron oxides with a crystalline core (Fe_3_O_4_) present great potential for use in oncological medicine due to their high compatibility [86], biodegradability [19] and easy synthesis [69]. Additionally, spherical magnetite nanoparticles with a diameter lower than 20 nm exhibit a superparamagnetic behavior that is a very important quality in medical imaging. An iron superparamagnetic particle (SPION) is made from a magnetite magnetic core, which provides the property of contrast in magnetic resonance imaging (MRI). This material has a biocompatible shell with a high number of functional groups, making it very useful for cancerous tumor follow up. Usually, a SPION is a magnetic single-domain structure, and it has high magnetic susceptibility. When such a special nanoparticle is placed in an alternating magnetic field, it can generate heat through Neel and Brown relaxation processes [87]. SPIONs do not have remanence and coercivity, as was explained in Section 4, and their agglomeration in human body is prevented.

Magnetic nanoparticles have received Food and Drug Administration (FDA) approval for the iron treatment deficiency, as a contrast agent in MRI and as a carrier for magnetic drug targeting therapy in different parts of the human body, biotherapeutical transport, magnetic hyperthermy, photodermal ablation, and photodynamic therapy [88].

Magnetic nanoparticles are also used in personalized medicine, termed theranostics. The diagnosis and monitoring of the cancer are performed based on medical imaging and through chemotherapeutical or biotherapeutical agents such as medicines, nucleic acids and proteins. A clear representation of the tumoral mass is very useful for doctors, because they can anticipate the patient’s response when a tumor is surgically removed and the future treatment is established. As medical imagining methods, X-ray radiography, echography, computerized tomography (CT scan), nuclear magnetic resonance (MRI) and positron emission tomography (PET) are analyzed.

The accumulation of magnetic nanoparticles at the tumor sites consists of two types: active or passive targeting. Passive targeting is based on the enhanced permeability and retention (EPR) phenomenon. A characteristics of the tumoral tissue represent the perfored blood vessels, and abnormal lymph drainage can be observed inside the tumor. Nanoparticles with dimensions lower than 100 nm can pass through these broken vessels, and they can migrate inside the tumor and remain there for a longer period of time, as compared with nanoparticle clearance in the blood (EPR phenomenon). Passive targeting is limited, because not all the cancerous tumors in the human body exhibit the EPR phenomenon and the permeability grade of the tumoral vascular system is not homogenous [89,90].

Active targeting is performed through nanoparticle modification, by attaching different groups to the MNP surface. These ligands have the possibility to recognize the biological structure’s unicity and neoplasm cells, which accumulate at the tumor site. The magnetic nanoparticles can be guided using an external magnetic field.

It has been proven that the iron oxide nanoparticles can generate microscopic holes inside endothelial cells. This phenomenon is called NanoEL. In order to not block the magnetic nanoparticles in the endothelial tissue, they are delivered through transcellular endocytosis or diffusion transport through the cellular membrane, because junctions between cells appear. The nanoparticles enter into vascularized organs such as the kidneys, liver or spleen at a high speed [91]. The NanoEL effect is much faster in comparison with endocytosis or diffusion through the cellular junction, and it is described in the existence of a intracellular link between the nanoparticle and VE-cadherin [92].

### 5.1. Cancer Cell Monitorization Using SPIONs

Today, the efficacity of the cancer treatment is estimated by taking into account the biopsy tissue analysis or using the medical images of the anatomical structures before and after the treatment. The time between the treatment’s initiation and the results’ apparition could be weeks or months. With the help of SPION nanoparticles, a real-time monitorization of the treatment is initiated, drugs are delivered, the response of the healthy tissue is evaluated, and the treatment adjustment becomes possible. A successful treatment was obtained in the case of murine dendritic cells with a combination of SPIONs, by increasing the anti-tumorigenic response through T cells stimulation. If dendritic cells are combined with SPIONs, it becomes possible to visualize their trajectories [93].

Other successful studies followed the pharmacokinetics of different active substances using SPIONs in different organs [94,95,96,97]. Another application of the SPIONs in drug delivery is made by detecting the changes in the two relaxivity parameters, T1 and T2. The transformation between a non-load state and a load state can be observed. In [98], studies were carried out in which the nanoparticles were used as a carrier for chemotherapeutical medicines, such as doxorubicin combined with Flutax 1 and DiR. The magnetic nanoparticles have a polymeric pH shell that degrades when an acid medium is present. The iron oxide carriers are called “nanophores”, and the loading with different active substances increases the SPIONs’ transverse T2 and longitudinal T1 nuclear magnetic resonance proton relaxation times. This type of chemotherapeutic treatment has proven to be more effective than classical routes, because the medicines do not have any chemical modification, and they are directly delivered at the tumor site. Drugs such as doxorubicin were modified and combined with polymers and targeting moieties, using the EPR effect. Nutrient receptors such as folate or transferrin receptors were also added to improve the drug delivery. Fluorochrome was used to trace the distribution of molecular groups. Doxorubicin was delivered in liposomal formulations, and clinical studies proved that higher doses of medicine could be released at the tumor sites, without many significant side effects. Ferumoxytol was approved by the FDA in 2009 to treat iron deficiency. In [98], these MNPs were analyzed as carriers for hydrophobic drugs, which were loaded inside the nanoparticle through the co-incubation process. This innovative delivery system could be approved faster by the FDA, because ferumoxytol is already accepted, and thus developing a way to load the active substances in a clinically approved vehicle will soon be easier in clinical studies. A great advantage of using magnetic nanoparticles consists of the fact that these materials could be followed through MRI and changes in relaxivity parameters.

Ferumoxytol is considered an ideal nanophore in drug delivery, and it was coated with carboxymethyl dextran [99]. After the dialysis process, performed in order to remove accidental cargo substances, the group was loaded with Flutax 1, which is a fluorescent taxol that can link to the taxol microtubule, a site with high affinity. It has been proven that ferumoxytol can also carry a near-infrared fluorophore DiR and the chemoterapeutical active substance doxorubicin. Through the dynamic light scattering method, it was observed that medicine loading has no effect on the average diameter of ferumoxytole, proving that no interactions occur between the nanoparticles and their cargo substances. The prepared colloidal solution has to be stable in a physiological medium (i.e., basic pH), and when the acidic conditions are met, the active substance is released. Ferumoxytol keeps doxorubicin at a pH of 7.2, and then, since pH decreases, it slowly releases the drug [98].

Loading or unloading the iron oxide particles can be observed, without using fluorophores, by analyzing the changes in T2 and T1 parameters and using an MRI device. Slight changes in these parameters were observed at a pH of 6.8. As the pH decreased, rapid decreases were also measured for T2 and T1, suggesting that the active substance is released at the tumor site.

### 5.2. Chemotherapy

**Figure 13:**
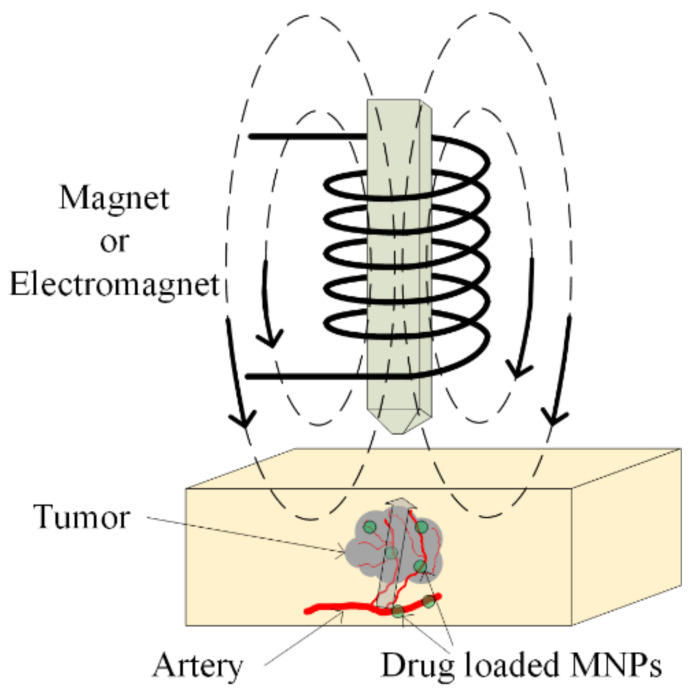
Magnetic drug targeting application. Drug-loaded MNPs are directly injected in the arterial blood of the tumor. A magnetic field is applied, and it causes MNPs’ enhancement in the tumoral zone.

The main aim of chemotherapy treatment is to avoid tumor growth with the help of chemical agents, which inhibit cellular function. The DNA chain can be interrupted by using these agents, and repair of the DNA is prohibited. Sometimes, different types of proteins can be administrated to stop the multiplication of damaged cells [100]. The chemotherapeutical drugs do not demonstrate selectivity, and they exhibit a high grade of toxicity in the human body, even acting on healthy cells. To avoid this activity, chemotherapeutic medicines are loaded in magnetic nanoparticles and directly delivered, as was already presented in Section 5.1 at the tumor site (see Figure 13). In this way, the overall toxicity of the treatment is decreased, and the active substance does not demonstrate chemical or physical degradation. In recent studies, TMZ, doxorubicin, paclitaxel and 5-fluorouracil were combined with SPIONs [101,102,103].

The alkylating agent temozolomide (TMZ) is an imidazotetrazine derivative of dacarbazine and the drug Temodar. TMZ has a lipophilic structure, and it can be orally administrated, because the human blood–brain barrier (BBB) is permeable to this substance. The compound presents stability at a pH lower than 5 and at pH higher than 7 if it is hydrolyzed [104].

Temozolomide was approved in 2005 to treat glioblastoma (GMB) patients. Glioblastoma multiforme is grade IV astrocytoma cancer [105]. Firstly, studies were conducted in which a classical treatment approach was taken into consideration. In the first phase of these clinical studies, the patients were administered drugs at doses between 750 and 1000 mg/m^2^, 5 days a week for 4 months. TMZ was combined with radiotherapy and the GBM patients took 75 mg/m^2^/day for 6 weeks, and at the same time focal radiotherapy was applied. Then, they took 150 mg/m^2^ TMZ 5 days/week followed by at least 20 days free of cancer treatment [106].

Due to the high toxicity of this drug, poor TMZ penetration across BBB, short half-life, the non-specific nature of the active substance and its non-targeted character, it is be better to functionalize it with different ligands (i.e., folic acid) and then load it in magnetite nanoparticles coated with a polymeric shell, composed of poly(ethylene glygol)-poly(butylene adipate)-poly(ethylene glycol) (TMZ-SPION-PEG-PBA-PEG) [107].

This shell, formed from tree layers of biodegradable and biocompatible polymers, helps to improve the therapeutic efficacity. This hydrophilic shell inhibits the reticuloendothelial system’s capacity to recognize and eliminate the MNPs. A higher stability in the physiological fluids observed, and increased blood circulation is obtained. The magnetite hydrophobic core is suitable to be loaded with lipophilic medicines, and their release at the tumor site is controlled.

The magnetic nanoparticles have dimensions between 10 and 100 nm, and thus the accumulation of SPIONs at the tumor zone is performed via the passive targeting process, as well as the EPR effect. Folic acid (FA) can be linked to the folate receptor (FR), which is present in cancerous cells such as GBM, breast and ovarian unhealthy tissues [107].

Another way to prepare an efficient treatment for GBM was proposed in [108], by preparing lipid magnetic nanovectors (LMNVs) loaded with SPIONs and functionalized with transferrin receptor antibodies (anti-TfR Ab). An outer shell made from biotin and m-PEG was analyzed, exploiting a biotin–streptavidin interaction.

Another study described aminosilane-coated SPIONs and took into account the use of magnetic hyperthermia to treat the GBM [109]. If a SPION is loaded with TMZ, its efficacity is increased by using a chlorotoxin (CTX), which that has a beneficial effect on the final product’s stability [110].

Some studies [111,112], in the case of bladder cancer, have shown that the treatment with mitomicyn-C decreased the recurrence rate after 10 years by 40%.

### 5.3. Immunotherapy

The immunotherapy method is an old one, but classical treatments are effective only in a small group of patients. Factors that influence the treatment success are previous treatments, the heterogeneity of the tumor and its stage, and the immunosuppressive phenotype of the cancer [113]. A higher grade of immunogenicity of the tumor ensures a better response to the immunotherapy. This type of therapy increases the immune system response to the cancerous tumor, by stimulating it or by hindering the cancerous cell signal. Artificial antibodies were developed that fight against the tumor, using different routes, such as antigens (Herceptin-targeting HER-2/neu for breast cancer), antibodies, which sever the blood supply of the tumor, cytokines such as interferon α and β and immune inhibitors (PD-1 inhibitors) [114,115].

Superparamagnetic nanoparticles are used together with immunotherapy, and the particle dimensions are very important. SPIONs with dimensions lower than 1000 nm are used for liver treatments, and particles smaller than 10 nm can be filtered by the kidneys. Due to lympho-hepatic tropism, SPIONS with intermediate dimensions can form agglomerations in the spine, liver or spleen, and in lymph nodes. These nanoparticles demonstrate an interaction with macrophage monocytes, dendritic cells and tumoral cells, which can be embedded through phagocytosis or micropinocytosis, depending on the surface nature [116,117].

The macrophage cells are phagocytic cells of the innate immune system. They represent the first response of the human body to infections, acting in pathogen recognition, the degradation of cellular intruders, and phagocytosis. Macrophages work well in the presence of T cells, and they induce tissue homeostasis, repair, and reconstruction of the damaged tissue. They also have a great disadvantage, because they are usually associated with autoimmune disease, cancers, angiogenesis, metastasis, and tumor development [118].

Dendritic cells are also a cellular line that detect the existence of pathogens and play an important role in the activation of the immune system. They have antigenic peptides on the major histocompatibility complex (MHC), and induce T-cell activation and differentiation. Dendritic cells are usually called sentinel cells, and they are located inside the lymphoid organs or at interfaces such as the intestines or skin [119].

The human immune system has different mechanisms for transport and integration, in which the magnetic nanoparticles are used in tumor treatment and differential medical imaging. A particular case is lymphatic drainage, realized using magnetic nanoparticles. MNPs are delivered in the lymphatic system, or they are transported by the antigen cells and guided through the lymphatic system to lymph nodes, where MNPs localize in different types of cells, and can regulate the immune system’s response. In order to increase the embedding process of the nanoparticles, different types of receptors and antibodies can be linked. Antibodies were used which control the epithelial cells’ growth for the encapsulation of the carcinoma cells [120]. If the corresponding cells are not vital for the human body, the antigen differentiation process can be successfully applied.

The T anticancer cells are considered indispensable tools for the eradication of different types of neoplasm. If, at the tumor site, an increased number of T cells is detected, the survival rate of the patient increases. T cells have receptors that can recognize pathological cells at the tumor site. By blocking the proteins CTLA-4 and PD-1 through specific antibodies, which are present in drugs, they can restore the entire immune system [121,122,123,124]. Pembrolizumab recently received FDA approval for different types of cancers, such as esophageal or gastroesophageal carcinoma, melanoma, applicable for patients with inoperable tumors or metastatic cases, non-small cell lung cancer, small cell lung cancer, head and neck squamous cell cancer, classical Hodgkin Lymphoma, etc. This drug is a monoclonal antibody directed against the PD-1 receptor on the cell surface, and it prevents the binding and activation of PD-L1 and PD-L2 and activates the T cells. Another monoclonal antibody is nivolumab, and it received FDA approval in 2015. Its action route is similar to pembrolizumab, and it can be combined with different chemotherapeutic approaches. It is very suitable for use with MNPs, but the PD-1 monoclonal antibodies target ligands and they can break the link between PD-L1 on cancerous cells and the immune system [125,126]. The first experiments consisted of loading the T cells with SPIONS as vehicles for immune modulatory substances and injecting and guiding them into the tumor sites by applying an external magnetic field [125]. Magnetic nanoparticles have an influence on macrophage polarization modulation, because it is already well known that the macrophage monocytes present two polarization states: non-tumorigenic M1 and pro-tumorigenic M2. In the case of a human monocyte line, it is possible to transform the phenotype M2 into phenotype M1, when SPIONs are used [126,127,128,129,130,131,132,133,134,135,136,137,138,139,140].

### 5.4. Radiotherapy

Radiotherapy is one of the most important treatments used to fight cancer. Tumor radiosensitization is an important phenomenon, and it can be enhanced by combining classical radiotherapy with different drugs or even with magnetic nanoparticles. Magnetic nanoparticles present a high value of the atomic number Z and exhibit important X-ray photon absorption, leading to an increase in secondary electrons that produce human DNA deterioration when the radiation dose is increased [141]. Nanoparticles, which are characterized by a high proton number, are usually linked to increased oxidative stress, expressed by reactive oxygen species (ROS) such as hydroxyl radicals that damages the cells’ DNA.

SPIONs were used for a long time as contrast agents in MRI investigations, but they increase the radiosensitivity of the tumor, making the radiotherapy treatments more effective [142,143,144]. In [145], it was found that the radiosensitizing properties of SPIONs have an influence on the MCF7 breast cancer cells, a fact that was indicated by the production of reactive oxygen species. This effect was analyzed in [146,147] in the case of colon cancer cells, in which SPIONs were combined with a 150 MeV proton beam. When 13 nm iron oxide nanoparticles were concomitantly used with 7.1 keV monochromatic X-rays, the colon tumor cells in murine animal model were drastically reduced [148]. In the radiotherapy treatment domain, energy sources in the MeV range are used, and the Compton effect is present [149].

**Figure 14:**
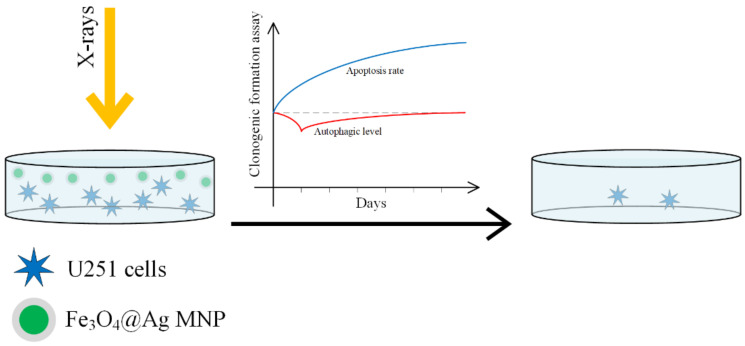
Fe_3_O_4_@Ag nanoparticles acting as a nano-radiosensitizer for GBM cellular culture treatment. The MNP radiosensitivity magnification effect is due to a decrease in the autophagy at the beginning of the treatment and followed by an increase in the apoptosis.

In [150], the radiosensitizing efficacity of Fe_3_O_4_–OA, Ag and Fe_3_O_4_@Au nanoparticles was compared on a U251 cellular line (GBM line). A schematic representation of the process is presented in Figure 14.

When SPIONs functionalized with gold or silver are used, the surface properties are changed. The gold nanoparticles increase the cellular death rate due to radiation, by interacting with thiols in the cytoplasm. Silver nanoparticles increase radiosensitivity in glioma by inducing cell apoptosis. This enhancement is stronger than in the case of gold nanoparticles [151,152,153,154,155]. SPIONs are guided to the tumor sites with the help of an external magnetic field, they can produce an increase in the local temperature due to magnetic hyperthermia, and through a Fenton reaction they can lead to ROS production. The best results were obtained in the case of Fe_3_O_4_@Ag, and it can be concluded that this material has a highly efficient radiosensitivity that adds to the radiation effect and destroys the cancer cell. The classical cell death processes due to radiation are necrosis, apoptosis, and autophagy [156].

Some studies have reported that a combination between growth factor receptor (C225) and Fe_3_O_4_@Ag can increase the sensitivity of nasopharyngeal carcinoma cells to radiotherapy, and the radiosensitivity of Fe_3_O_4_@Ag is higher than gemcitabine (Gemzar) or 5-fluoroacil [157].

In [158], 38 clinical studies were compared, obtained between 1987 and 2014. This clinical research took into account different types of neoplasm, such as head, neck, esophagus, skin, lungs, etc. Radiotherapy was applied as a singular treatment to 1717 persons, and a combined treatment of radiotherapy and hyperthermy (thermoradiotherapy) was used for 1761 patients. In these cases, an increased rate of survival of 54.9% was recorded, as compared with the rate of 39.8% that was determined for single therapy. The hyperthermic effect of SPIONs increases the radiotherapy’s effect [158,159].

### 5.5. Magnetic Hyperthermia

**Figure 15:**
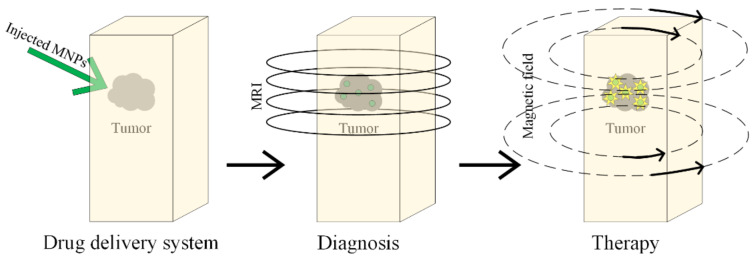
Diagnosis and treatment of the tumor based on MNPs, using magnetic hyperthermia.

Magnetic hyperthermia has been known about since 1957, and it was first presented in [160], where cancerous tumors were heated with the help of magnetic particles placed in an external magnetic field (Figure 15).

When magnetic nanoparticles are mediated with magnetic hyperthermia, the treatment is very efficient, because a lot of cancerous cells are killed, and the surrounding healthy tissues are left undamaged [160,161,162,163,164,165]. This combined treatment is based on intracellular hyperthermia, and it is possible to heat only the harmful cells and the link between cells and targeting ligands with MNPs becomes possible. It can be observed that the local and homogenous heat presents a high grade of selectivity. This type of treatment is in clinical trials for GBM cancer [166].

Magnetic hyperthermia that uses MNPs or SPIONs (SPIONs-MH) is based on locally increasing the temperature above 42 °C, with an impact on cancer cell physiology, by altering the structural proteins and generating the necrosis or apoptosis of the cells [162,165,167].

The treatment’s success depends on the following factors: (1) the cancerous cells must be selectively targeted; (2) the SPIONs’ penetration inside the cancerous cells should be facile; (3) the encapsulation of the SPIONs in the unhealthy cells has to be completed; (4) SPIONs must be biocompatible and non-toxic; (5) the treatment should be feasible from the clinical point of view; (6) the treatment has to be a general one, and different types of tumors can be treated, even metastatic ones [168,169].

The SPIONs-MH have strict limitations regarding the effective value *H* and the frequency *f* of the alternating applied magnetic field, so the product *H* × *f* must be strictly lower than 5 × 10^9^ due to biomedical conditions [170]. When SPIONs are used, it must be taken into account that sometimes, their low thermal conversion efficiency is due to a decreased value of magnetic susceptibility, and this is an important problem in medical applications. In order to address this limitation and to obtain improved induction heating properties, modulation of the particle size [171], a strict control of the chemical composition [172,173,174,175,176,177,178,179], biofunctionalization of the SPIONs’ surface, and modification of the particle shape [86,180] should be taken into account. It is already well-known that an accumulation of SPIONs in lysosomes, which are known as the “suicidal bags” of the cell, because they can destroy their own cells through specific hydrolytic enzymes, leads to lysosomal cell death by increasing the local temperature and the production of ROS inside the lysosome [181,182].

The heating effect of SPIONs is due to the magnetic hysteresis phenomenon, Neel relaxation, induced eddy currents and Brownian relaxation. The time interval between two random flips of the SPION magnetization due to temperature is known as the Neel relaxation time. SPIONs are ferrimagnetic or ferromagnetic nanoparticles, because they contain iron, and so a small hysteretic dependence between magnetic flux density and magnetic field strength is expected to appear. The energy losses, necessary to magnetize such a material, are usually called hysteresis losses. The heat generated by SPIONs is equal to the hysteresis cycle area, and in the case of a very small nanoparticle, the magnetization of the material is due to spin magnetic moment rotations. When an alternating magnetic field is applied, the spins’ rotation must overcome the friction phenomenon, which appears between the easy magnetization axis of the particles and atomic lattices due to the Neel relaxation phenomenon. The friction effect can also be generated between the SPIONs and the area near them in the case of Brownian relaxation. In nano-magnetism, the Neel and Brown relaxation mechanisms are classical processes which completely characterize the single-domain nanoparticle magnetization [77].

If the time necessary to measure the magnetization of the nanoparticle is higher than the Neel relaxation time, in the absence of an external magnetic field, the average magnetization of SPIONs, dispersed in different aqueous solution, is equal to zero [183].

The main experimental technique used for measuring MNPs’ magnetic heating is calorimetry, which measures the specific heat absorption rate (SAR), specific loss power (SLP) and intrinsic loss power (ILP) [181,184]. The hysteresis loop of SPIONs or MNPs is determined through alternating current magnetometry. The heat dissipated during a cycle is equal to the hysteresis area, and SAR can be computed as in [171,181], but this type of experimental measurement is performed at different values for the magnetic field strength and frequency that are not in the biological range, and so the magnetic characterization is not entirely accurate for SAR detection [185]. In Table 3, experimental data, obtained in the case of Fe_3_O_4_ magnetic nanoparticles with different shape and sizes, are presented [184,185].

When the shell of an MNP has been functionalized with a fluorophore through a bond, which can break at a given temperature [186], it is possible to measure the fluorophore content after the heating process, and the bond breaking temperature is determined through fluorescence thermometry.

In the case of the application of an alternating magnetic field, the MNPs develop due to the Joule effect and eddy current losses, which contribute to an increase in the total heating effect. In [187], the thermogenesis of MNPs when an external magnetic field is applied was presented. Films of Au@MNPs present a magnetothermal effect at a high frequency in the order of 10^2^ kHz.

**Figure 16:**
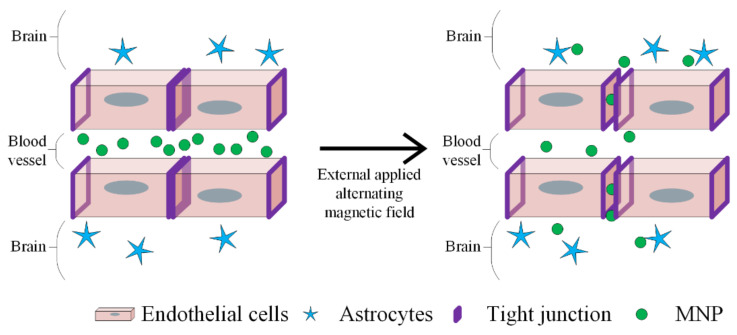
BBB disruption using MNPs and applying an alternative magnetic field. The heat generated by MNPs opens up the tight junction to deliver the drugs. The endothelial cells, astrocytes and MNPs are represented by different symbols, as indicated in the figure legend.

MH is a promising therapeutic strategy in GBM treatment, as depicted in Figure 16.

SPIONs can be coated with aminosilane, a polymer that improves protein and cell adhesion. This material was used in preclinical investigations [188,189,190] and in some phase I and II clinical trials [191,192,193,194,195,196]. The first clinical study was described in [195], and it was applied in the case of 14 GBM patients. The entire group received a 15 nm aminosilane-coated MNP suspension that was neuro-navigationally guided through the tumor site, followed by 4 to 10 thermotherapy applications. At a maximum field value of 14 kA/m in 90% tumor volumes, a temperature between 39 and 46 °C was obtained.

In 2009, a second postmortem study on three GBM patients was developed, and histological analysis showed that MNPs were confined near the tumor sites, demonstrating that it is necessary to administer MNPs in multiple places in order to obtain a uniform heat production [193].

Another clinical study performed on 66 patients using MH in combination with radiotherapy was published in [196]. Although an increased survival rate was obtained, serious side effects such as increased intracranial pressure or necrosis [197] and moderate side effects such as headache, fatigue, thrombocytopenia, leukopenia, vomiting were reported.

The general characteristics of the SPIONs used in MH is that the SAR depends on the particle size, shape, magnetization, chemical composition, and concentration of SPIONs, magnetic field intensity and frequency. It is better that magnetic fields with high oscillation frequencies are avoided due to eddy current generation [198].

Magnetic hyperthermia mediated through SPIONs has proven its efficacity, by causing about 50% cell death of the U87-MG cellular line (GBM—malignant glioma) compared with only 20% cell death in the HUVEC line (healthy endothelial cells) [199]. Zinc-doped ferrite nanoparticles have a higher saturation point than in the case of classical particles and a constant anisotropy energy, and they have proven to be effective for U87-MG by inducing a hyperthermia effect for a frequency of 700 kHz at 1.7 kA/m [200]. The achieved temperature was 41.5°C, and it was high enough to produce cell death. Human-like collagen protein-coated SPIONs have a rapid heating capacity, by comparing them with uncoated particles, because the MNPs’ agglomeration is reduced, and they have an enhanced surface stability [194]. Another approach consists of using iron oxide-coated cationic liposomes, because they present a high cellular affinity and electrostatic interactions with the anionic phospholipids from the cellular membrane. Their effect was investigated on T-9 glioma cells, and a temperature of 42 °C was achieved in 20 min, and as a final result, 100% cellular cancerous death was obtained in 40 min [174,194,199,201,202]. In [194,203], poly-ethylene-glycol-based hydrogel with MNPs, which induces MH, was analyzed on M059K glioblastoma cells, and the results were satisfactory.

Magnetic hyperthermia has a great potential to become a first line treatment for GBM patients and for other cancers. Other studies have incorporated MNPs and methotrexat (MTX) to treat breast cancer [159,204,205,206]. Other chemotherapeutical medicines, such as doxorubicin, can be combined with MH treatments, making this approach more effective. Another advantage consists of the damage to the extracellular matrix of cancer cells, due to heat effect that facilitates drug elution at the tumor site. These types of studies were performed on a murine animal model.

### 5.6. Gene Therapy

Gene therapy is similar to chemotherapy, and the main difference between them is that instead of delivering small drugs, guided biological agents such as DNA, RNA or siRNA, proteins, and peptides are delivered to the tumor site, and in this way the cellular death can be induced. When cancer appears in one body part, the deteriorated human DNA is characterized through the appearance of atypical proteins, and the disease can be treated through defect gene replacement inside the cancerous cells. Alternatively, siRNA works by suppressing the protein actions, which are due to the cancerous cells. As a classical definition, siRNA is a double-stranded interfering RNA formed from a 21–23-nucleotide sequence. siRNA is combined with nanoparticles that can carry and deliver it to cells, because medical RNA has the property to be very degradable when it is directly injected into the bloodstream [207]. From siRNA, RNA-induced silencing complex (RISC) is produced, which is formed in the cell cytoplasm. The RISC binds the mRNA of the cell. siRNA is usually used to target oncogenes. When siRNA is carried by an MNP, the particle becomes almost unrecognizable for the human body, the cell uptake will be improved, and the circulation time becomes longer. The MNPs will accumulate at the tumor sites and due to tumor vascularization, the particle can easily enter inside the tumor, and the poor lymphatic drainage permits particle accumulation.

The magnetic nanoparticles, which encapsulate or bind DNA or RNA, are covered with a cationic polymer, leading to a positively charged layer, favoring an electrostatic interaction between negatively charged nucleic acid and the MNPs. Other studies showed that for a successful DNA delivery, it is necessary that the MNPs interact with the cell nucleus [208,209]. Recent approaches involved the development of “intelligent” MNPs that use specific coatings, which prevent the delivery carrier from being degraded and permit drug release after entering the tumoral cell. Some initial coatings were performed with disulfide-containing polyethylimine (PEI), and in recent years, polymer shell coatings based on chitosan, PEI, and PEG were developed [210].

In the case of GBM cancer, the tumor presents many genetic aberrations, and cationic-coated MNPs as non-viral vehicles for gene drugs were able to deliver tumor necrosis factor-related apoptosis-inducing ligand (hTRAIL), encoding plasmid open reading frame (pORF-hTRAIL) with high efficiency and survival rate in a rodent glioma model [211]. In the near future, gene delivery with the help of MNPs could become a safe option to treat GBM, because MNPs represent a new way to address infiltrating cells of GBM.

### 5.7. Ferroptosis

Ferroptosis is classified as an oxidative regulated cell death (RCD), and the main involved mechanisms are iron-dependent peroxidation of lipids and plasma membrane rupture [212]. Inside the living cells, the mitochondria represent the most important reactive oxygen species (ROS) source. Ferroptosis can be carried out in two ways: extrinsic or intrinsic. The extrinsic modality consists of cell membrane transporters’ (cystine/glutamate) inhibition or by stimulating iron transporters such as serotransferrin and lactotransferrin [213]. The intrinsic pathway is characterized by a blocking of intracellular antioxidant enzymes such as glutathione peroxidase (GPX4). In the case of an increased intracellular amount of lipid reactive oxygen species (L-ROS) that exceed the GPX4, it results in damage to the cellular redox homeostasis [214].

Ferroptosis has three important characteristics, as follows: the oxidation reaction of polyunsaturated fatty acid (PUFA) from the phospholipids of the cellular membrane, the presence of iron as a result of redox chemical reaction, and finally the loss of lipid hydroperoxide’s (LOOH) repair properties [215]. It can be concluded that ferroptosis is usually due to a deadly metabolic imbalance, and it can represent an adequate solution for cancers which are resistant to other therapies [216]. Changes that appear in the cell mitochondria, such as volume decrease, bilayer membrane density increase, external mitochondrial membrane disruption, and the absence of mitochondrial cristae [217,218], are important parameters and favor the ferroptosis process. In [219], it was shown that if a clear and spherical cell which contains an empty cytosol is microscopically detected, is considered to be a ferroptotic cell.

From a biochemical point of view, the ferroptotic cell is characterized by the peroxidation of PUFAs in membrane phospholipide due to an increased quantity of redox active divalent iron Fe^2+^. This process is controlled through GPX4, which converts LOOH into a lipid alcohol [220].

Different genes connected with iron metabolism in the human body (transferrin receptor TFR1, iron response element binding protein 2, IREB2 and ferritin heavy chain FtH), lipid synthesis and oxidative stress pathways are also involved in controlling the ferroptosis process [221,222].

Ferroptosis can be considered an useful tool, because the FDA have approved the so-called ferroptosis inducers (erastin, sorafenib, sulfasalazine (SAS), cisplatin, 1-buthionine sulfoximine (BSO), artesunate (ART), lanperisone, RSL3, altretamine, iron ionophores, doxorubicin into mesoporous carbon nanoparticles/withaferin A/poly(ethylene glycol)-coated (PEGylated) silica nanoparticles, etc.).

SPIONs are very important in this RCD process, because they induce cellular death through an increase in iron level and ROS generation [223]. ROS include superoxide, oxygen peroxide, hydroxyl radical, singular oxygen species, peroxyl radical, alkoxyl radical, hypochlorous acid, and ozone. In [223], cisplatin with SPIONs as a carrier based on FePt had proven efficacity through the hydrolysis of iron oxide contained in SPIONs. Cancer cells have an acidic pH, which favors iron intracellular release and Fenton-like reaction generation, which produce ROS. The chemical Fenton-like reaction consists of divalent iron Fe^2+^’s reaction with hydrogen peroxide (H_2_O_2_), generating ROS [224,225,226].

**Figure 17:**
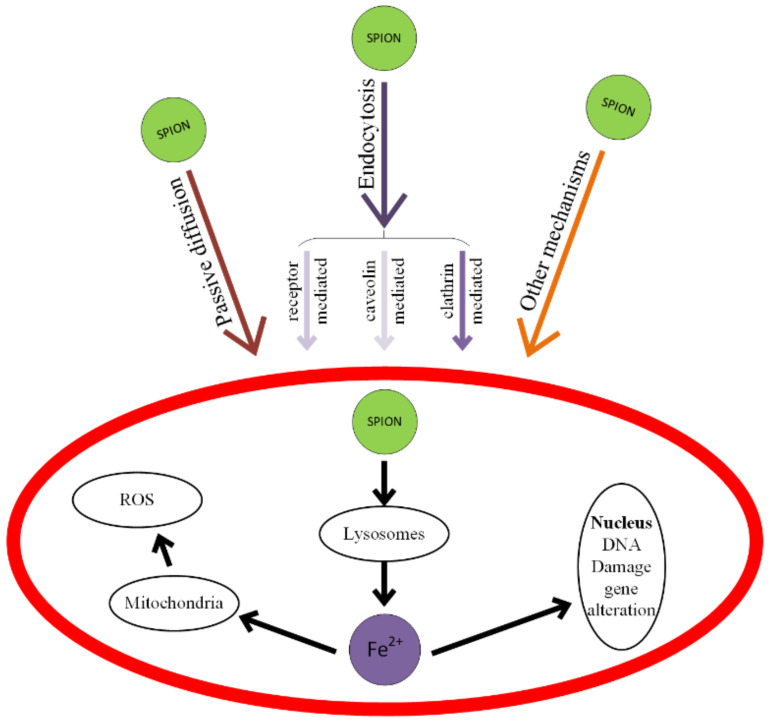
Mechanisms of SPIONs’ interaction with cells and types of induced toxicity. SPIONs mainly interact with cells through passive diffusion or endocytosis. The endocytosis pathways are: clathrin-mediated, caveolin-mediated and other receptor-mediated.

When SPIONs, considered as ROS generators through a Fenton-like reaction, are combined with anticancer drugs, the treatment will be more efficient. The toxicity of SPIONs is shown in Figure 17 and consists of cell membrane deterioration, cytoskeleton structural modifications, oxidative damage of DNA chains, lysosome working condition modifications, ROS generation, mitochondria damage, and the generation of inflammatory factors [227,228].

Mitochondria-mediated ferroptosis has a potential use in cancer treatments, but supplementary in vivo models to fulfill this theory are necessary at this moment of time [213,221,222,224,225,226,227].

## 6. Challenges and Discussions Regarding the MNPs Use in Cancer Therapy

Nanoparticles are used more and more oftne in many medical fields as orthopedy, stomatology and neurosurgery. In [229,230,231,232], composites consisting of a combination between magnetic nanoparticles, calcium phosphates and biopolymers are prepared through a biomimetic co-precipitation method for bone tissue engineering. In dental restauration, metallic nanoparticles are usually involved in designing adhesives used in coherence tomography evaluation. Graphene-based nanomaterials are another innovative solution for tissue engineering in the dental field [233,234,235]. Selenium nanoparticles were involved in surface modification of the titanium mesh for cranioplasty [236,237,238]. In the nanoparticle field, magnetic nanoparticles represent a special class, because they demonstrate an intrinsic association with oncological domain.

Although some MNPs and SPIONs have received FDA approval, their use as treatments in cancer therapy should be carefully conducted and controlled, because a high amount of these materials could be harmful. MNPs and SPIONs can exhibit toxicity, and thus they sometimes are functionalized with different inorganic or organic compounds in order to obtain a passivate surface. Modern vectors for drug-delivery applications are also silica-based mesoporous structured materials, due to their specific textural characteristics, consisting of uniform pores with laboratory-controllable sizes, a high specific surface area and high chemical and thermal stability [60,61]. Different studies, presented in the paper, provide evidence that MNPs have a very low level of toxicity, when their concentration in solutions is lower than 100 μg/mL. The analysis made in vivo or in vitro proves that SPIONs can alter living cells due to cytoskeletal deterioration, ROS generation, mitochondria damage, and DNA structure change. It is supposed that the SPIONs’ toxicity is not due only to their ferromagnetic or ferrimagnetic cores, but also to nanoparticle agglomerations, which can become instable in biological media or different solutions, similar to body fluids. In the case of SPIONs, the shape (spherical, rod or beam), the dimensions, the outer shell, and the iron/material ratio used for the outer shell (polymer or gold) are important parameters that must be taken into account.

If laboratory-developed magnetic nanoparticles are used in animal models, their toxicity has to be carefully analyzed. A high number of MNPs or SPIONs, FDA-approved or laboratory-developed, were successfully applied on murine animal models. In 2007, clinical studies were initiated regarding the SPIONs used in the treatment of GBM patients, through magnetic hyperthermia. If MNPs are addressed for clinical investigations, the physic-chemical properties, toxicity, and the possibility to become carriers for different drugs have to be considered. The shell of the magnetic core must be biocompatible, and the liver and kidneys have to synthetize them fast. The degraded components must also be smaller than 5 nm.

When SPIONs are used in magnetic hyperthermia, their distribution is uniform and the therapeutical temperature has to be achieved inside the tumor volume, and the surrounding tissue must present minimal damage [228].

MNPs and SPIONs have different side effects, such as liver, brain, skin, stomach function deterioration, with visible symptoms such as hair loss, nausea and vomiting and even nerve damage that could unfortunately be persistent for 10 years [213].

MNPs are monitored with the help of MRI techniques, and this is a very important step in cancer monitorization and drug delivery treatments. Today, MNPs are of great importance, because they are considered very useful in early-stage cancer detection, and in the near future, they may be a viable treatment for patients who suffer from aggressive types of cancer such as GBM. It is expected that this technology will applied at a medium scale for patients at a global level.

Some studies [213,221,222,224] claim that the side effects of the MNPs used in chemotherapy, radiotherapy, gene therapy, immunotherapy and magnetic hyperthermia can be successfully addressed by the self-defense system of the body.

The most important characteristic of the MNPs is that they can be linked to drugs, antibodies, proteins, enzymes and can directly target a specific organ or tumor site using an external magnetic field. MNPs or SPIONs can be heated in alternating magnetic fields and they can destroy the cancer cells.

An important collaborative work between medical specialists and nanomaterial science researchers is important, because together, they can generate steps forwards to ameliorate or cure cancer. As future perspectives, important progress has been made in the development of new MNPs for pre-clinical cancer theranostics applications. Notable steps were made in the synthesis of efficient magnetite/mesoporous media as drug delivery treatments, and in the near future, complex platform could be developed for cancer treatments [60,61,239]. Few nanomaterials have shown success in clinical cases, because supplementary research must be carried out in order to understand nanomaterial–human cell interactions and biological barriers in different types of cancers [240,241]. There are also industrial barriers because good laboratory practice has to be developed. An increase in the number of preclinical trials with MNPs has been observed, and it is expected that this will evolve into clinical studies, by taking into account the great potential of MNPs to diagnose and treat cancer. The in vivo behavior of MNPs must be carefully investigated. In order to obtain a high efficiency of MNP treatments, a size reduction and the use of different bio-compatible shells have to be taken into account. In this way, the blood circulation and the time taken to reach the cancerous tissues is greatly reduced. As we have underlined in the paper, despite MNPs’ success in murine models as theranostic agents, their loading capacity and their affinity to target cancer cells must be analyzed and improved if possible. The topic of MNPs is a new and important research domain, which is in continuous development, and synthesis of magnetic drug delivery systems should be investigated. Two very important future perspectives consist of MNPs’ elimination from the living body and overcoming the reported long-term toxicity cases.

## Figures and Tables

**Table 1:** Synthesis methods for magnetic nanoparticles.

	Synthesis Method	Conditions	Size (nm)	Particle Morphology	Reference
Chemical methods	Aqueous co-precipitation	Temperature 20–90 °C	15–200	Spherical, rhombic	[24]
		Water solvent			
	Sol-gel synthesis	Temperature 200–400 °C	20–200	Spherical	[23]
		Organic solvent			
	Hydrothermal synthesis	Temperature 220 °C	520	Spherical	[25]
		Water-ethanol solvent			
	Microemulsions	Temperature 20–50 °C	4–12	Spherical, cubic	[26]
		Organic solvent			
	Electrochemical deposition	Temperature 70–100 °C	3–8	Spherical	[22]
		Organic solvent			
	Polyol method	Temperature 120–280 °C	5–40	Spherical	[22]
		Organic solvent			
Physical methods	Sonochemical method	Temperature 25 °C	10–30	Spherical, rod	[22]
		Water			
	Thermal decomposition	Temperature 100–320 °C	3–20	Spherical	[25]
		Organic			
	Spray pyrolysis	Temperature 400–700 °C	5–60	Spherical with aggregates	[27]
		Organic			
	Laser-induced pyrolysis	Temperature 1100 °C	5–30	Spherical less large	[27]
		Organic			
Biological methods	Biomimetic synthesis	-	50–100	Spherical cluster,	[28]
				Cubo-octahedral	
	Microbial synthesis	Virus Tobacco mosaic virus	5	Disordered, prismatic and film	[28,29]
	Plant-mediated synthesis	Eucalyptus Leaf	20–80	Spheroidal	[28]

**Table 2:** Main magnetic properties of Fe_3_O_4_ nanoparticles.

Synthesis Method	Size (nm)	*M_s_* (emu/g)	*H_c_* (Oe)	Reference
Aqueous co-precipitation	4.9	60		[81]
	6.3	65		
	8.6	58		
Thermal decomposition	4.2	75	318	[82]
	7.4	70	270	
	8.1	65	70	
	17	82	364	
	45	92	640	
Non-aqueous homogeneous solution of polyols	6.6	71	16	[83]
	11.6	77	15	
	17.8	83	3	

**Table 3:** Shape and size effect on MNPs’ magnetic heating parameters for Fe_3_O_4_ nanoparticles [184,185].

Shape	Size (nm)	*f* (kHz)	*M_s_* (emu/g)	*H* (Oe)	SAR/SLP (W/g)	ILP (nHm^2^/kg)
sphere	22	500	65	195	716	5.96
disc	125	488	435	600	5000	4.48
ring	70	400	-	744	3050	2.18

## Data Availability

Data sharing not applicable.

## References

[1] Seo W., Lee J., Sun X., Suzuki Y., Mann D., Liu Z., Terashima M., Yang P.C., McConnel M., Nishimura D. (2006). FeCo/graphitic-shell nanocrystals as advanced magnetic-resonance-imaging and near-infrared agents. Nat. Mater..

[2] Laurent S., Saei A.A., Behzadi S., Panahifar A., Mahmoudi M. (2014). Superparamagnetic iron oxide nanoparticles for delivery of therapeutic agents: Opportunities and challenges. Expert Opin. Drug Deliv..

[3] Cornell R.M., Schwertmann U. (2003). The Iron Oxides: Structure, Properties, Reactions, Occurrences and Uses.

[4] Ahmad T., Bae H., Rhee I., Chang Y., Jin S.U., Hong S. (2012). Gold-coated iron oxide nanoparticles as a T2 contrast agent in magnetic resonance imaging. J. Nanosci. Nanotechnol..

[5] Zhu N., Ji H., Yu P., Niu J., Farooq M.U., Akram M.W., Udego I.O., Li H., Niu X. (2018). Surface Modification of Magnetic Iron Oxide Nanoparticles. Nanomaterials.

[6] Ghorbani M., Hamishehkar H., Arsalani N., Entezami A.A. (2016). A novel dual-responsive core-crosslinked magnetic-gold nanogel for triggered drug release. Mater. Sci. Eng. C Mater. Biol. Appl..

[7] Lo C.K., Xiao D., Choi M.M.F. (2007). Homocysteine-protected gold-coated magnetic nanoparticles: Synthesis and characterization. J. Mater. Chem..

[8] Hu Y., Meng L., Niu L., Lu Q. (2013). Facile Synthesis of Superparamagnetic Fe_3_O_4_@polyphosphazene@Au Shells for Magnetic Resonance Imaging and Photothermal Therapy. ACS Appl. Mater. Interfaces.

[9] Hoang Thi T.T., Nguyen Tran D.H., Bach L.G., Vu-Quang H., Nguyen D.C., Park K.D., Nguyen D.H. (2019). Functional Magnetic Core-Shell System-Based Iron Oxide Nanoparticle Coated with Biocompatible Copolymer for Anticancer Drug Delivery. Pharmaceutics.

[10] Kumari P., Ghosh B., Biswas S. (2016). Nanocarriers for cancer-targeted drug delivery. J. Drug Target.

[11] ud Din F., Aman W., Ullah I., Qureshi O.S., Mustapha O., Shafique S., Zeb A. (2017). Effective use of nanocarriers as drug delivery systems for the treatment of selected tumors. Int. J. Nanomed..

[12] Yalcin S., Unsoy G., Mutlu P., Khodadust R., Gunduz U. (2014). Polyhydroxybutyrate-Coated Magnetic Nanoparticles for Doxorubicin Delivery: Cytotoxic Effect Against Doxorubicin-Resistant Breast Cancer Cell Line. Am. J. Ther..

[13] Fazilati M. (2014). Anti-neoplastic Applications of Heparin Coated Magnetic Nanoparticles Against Human Ovarian Cancer. J. Inorg. Organomet. Polym. Mater..

[14] Kostevšek N. (2020). A Review on the Optimal Design of Magnetic Nanoparticle-Based T2 MRI Contrast Agents. Magnetochemistry.

[15] Persano S., Das P., Pellegrino T. (2021). Magnetic Nanostructures as Emerging Therapeutic Tools to Boost Anti-Tumour Immunity. Cancers.

[16] Hajal C., Campisi M., Mattu C., Chiono V., Kamm R.D. (2018). In vitro models of molecular and nano-particle transport across the blood-brain barrier. Biomicrofluidics.

[17] Barua S., Mitragotri S. (2014). Challenges associated with Penetration of Nanoparticles across Cell and Tissue Barriers: A Review of Current Status and Future Prospects. Nano Today.

[18] D’Agata F., Ruffinatti F.A., Boschi S., Stura I., Rainero I., Abollino O., Cavalli R., Guiot C. (2018). Magnetic Nanoparticles in the Central Nervous System: Targeting Principles, Applications and Safety Issues. Molecules.

[19] Sun C., Lee J.S., Zhang M. (2008). Magnetic nanoparticles in MR imaging and drug delivery. Adv. Drug Deliv. Rev..

[20] Ebadi M., Bullo S., Buskaran K., Hussein M.Z., Fakurazi S., Pastorin G. (2021). Dual-Functional Iron Oxide Nanoparticles Coated with Polyvinyl Alcohol/5-Fluorouracil/Zinc-Aluminium-Layered Double Hydroxide for a Simultaneous Drug and Target Delivery System. Polymers.

[21] Hayashi K., Tokuda A., Nakamura J., Sugawara-Narutaki A., Ohtsuki C. (2020). Tearable and Fillable Composite Sponges Capable of Heat Generation and Drug Release in Response to Alternating Magnetic Field. Materials.

[22] Hosu O., Tertis M., Cristea C. (2019). Implication of Magnetic Nanoparticles in Cancer Detection, Screening and Treatment. Magnetochemistry.

[23] Chen Z., Wu C., Zhang Z., Wu W., Wang X., Yu Z. (2018). Synthesis, functionalization, and nanomedical applications of functional magnetic nanoparticles. Chin. Chem. Lett..

[24] Cristea C., Tertis M., Galatus R. (2017). Magnetic nanoparticles for antibiotics detection. Nanomaterials.

[25] Xu J.K., Zhang F.F., Sun J.J., Sheng J., Wang F., Sun M. (2014). Bio and nanomaterials based on Fe_3_O_4_. Molecules.

[26] Liu C., Zou B., Rondinone A.J., Zhang Z.J. (2000). Reverse micelle synthesis and characterization of superparamagnetic MnFe_2_O_4_ spinel ferrite nanocrystallites. J. Phys. Chem. B.

[27] Abd Elrahman A.A., Mansour F.R. (2019). Targeted magnetic iron oxide nanoparticles: Preparation, functionalization and biomedical application. J. Drug Deliv. Sci. Technol..

[28] Ahmad F., Ashraf N., Ashraf T., Zhou R.B., Yin D.C. (2019). Biological synthesis of metallic nanoparticles (MNPs) by plants and microbes: Their cellular uptake, biocompatibility, and biomedical applications. Appl. Microbiol. Biotechnol..

[29] Karade V.C., Dongale T.D., Sahoo S.C., Kollu P., Chougale A.D., Patil P.S., Patil P.B. (2018). Effect of reaction time on structural and magnetic properties of green-synthesized magnetic nanoparticles. J. Phys. Chem. Solids.

[30] Massart R. (1981). Preparation of aqueous magnetic liquids in alkaline and acidic media. IEEE Trans. Magn..

[31] Montaseri H., Alipour S., Vakilinezhad M.A. (2017). Development, evaluation and optimization of superparamagnetite nanoparticles prepared by co-precipitation method. Res. Pharm. Sci..

[32] Slimani S., Meneghini C., Abdolrahimi M., Talone A., Murillo J.P.M., Barucca G., Yaacoub N., Imperatori P., Illés E., Smari M. (2021). Spinel Iron Oxide by the Co-Precipitation Method: Effect of the Reaction Atmosphere. Appl. Sci..

[33] Hollingsworth J.A., Klimov V.I. (2010). Soft chemical synthesis and manipulation of semiconductor nanocrystals. Nanocrystal Quantum Dots.

[34] Shouheng S., Murray C.B., Weller D., Folks L., Moser A. (2000). Monodisperse FePt Nanoparticles and Ferromagnetic FePt Nanocrystal Superlattices. Science.

[35] Park J., An K., Hwang Y., Park J.G., Noh H.J., Kim J.Y., Park J.H., Hwang N.M., Hyeon T. (2004). Ultra-large-scale syntheses of monodisperse nanocrystals. Nat. Mater..

[36] Hyeon T. (2003). Chemical synthesis of magnetic nanoparticles. Chem. Commun..

[37] Choi J.S., Jun Y.W., Yeon S.I., Kim H.C., Shin J.S., Cheon J. (2006). Biocompatible heterostructured nanoparticles for multimodal biological detection. J. Am. Chem. Soc..

[38] Sanchez-Dominguez M., Pemartin K., Boutonnet M. (2012). Preparation of inorganic nanoparticles in oil-in-water microemulsions: A Soft and Versatile Approach. Curr. Opin. Colloid Interface Sci..

[39] Lopez-Quintela M.A. (2003). Synthesis of nanomaterials in microemulsions: Formation Mechanisms and Growth Control. Curr. Opin. Colloid Interface Sci..

[40] Song M.M., Song W.J., Bi H., Wang J., Wu W.L., Sun J., Yu M. (2010). Cytotoxicity and cellular uptake of iron nanowires. Biomaterials.

[41] Dippong T., Levei E.A., Cadar O. (2021). Recent Advances in Synthesis and Applications of MFe2O4 (M = Co, Cu, Mn, Ni, Zn) Nanoparticles. Nanomaterials.

[42] Jalalian M., Mirkazemi S.M., Alamolhoda S. (2016). The effect of poly vinyl alcohol (PVA) surfactant on phase formation and magnetic properties of hydrothermally synthesized CoFe_2_O_4_ nanoparticles. J. Magn. Magn. Mater..

[43] Mulens V., Morales M.P., Barber D. (2013). Development of magnetic nanoparticles for cancer gene therapy: A comprehensive review. Int. Sch. Res. Not..

[44] Morel A.L., Nikitenko S.I., Gionnet K., Wattiaux A., Lai-Kee-Him J., Labrugere C., Chevalier B., Deleris G., Petibois C., Brisson A. (2008). Sonochemical approach to the synthesis of Fe(3)O(4)@SiO(2) core-shell nanoparticles with tunable properties. ACS Nano.

[45] Lee D.C., Mikulec F.V., Pelaez J.M., Koo B., Korgel B.A. (2006). Synthesis and magnetic properties of silica-coated FePt nanocrystals. J. Phys. Chem. B..

[46] Chen X., Klingeler R., Kath M., El Gendy A.A., Cendrowski K., Kalenczuk R.J., Borowiak-Palen E. (2012). Magnetic silica nanotubes: Synthesis, drug release, and feasibility for magnetic hyperthermia. ACS Appl. Mater. Interfaces.

[47] Gutsch A., Muhlenweg H., Kramer M. (2005). Tailor-Made Nanoparticles via Gas-Phase Synthesis. Small.

[48] Kodas T.T., Hampden-Smith M. (1999). Aerosol Processing of Materials.

[49] Patel T., Zhou J., Piepmeier J.M., Saltzman W.M. (2012). Polymeric nanoparticles for drug delivery to the central nervous system. Adv. Drug Deliv. Rev..

[50] Liu D., He C., Poon C., Lin W. (2014). Theranostic nanoscale coordination polymers for magnetic resonance imaging and bisphosphonate delivery. J. Mater. Chem. B.

[51] Silva S.M., Tavallaie R., Sandiford L., Tilley R.D., Gooding J.J. (2016). Gold coated magnetic nanoparticles: From preparation to surface modification for analytical and biomedical applications. Chem. Commun..

[52] Kumar S., Gandhi K.S., Kumar R. (2007). Modeling of formation of gold nanoparticles by citrate method. Ind. Eng. Chem. Res..

[53] Mills A., Britton M. (2017). NMR study of the influence of n-alkanol co-surfactants on reverse micelles in quaternary microemulsions of cetyltrimethylammonium bromide (CTAB). Magn. Reson. Chem..

[54] Dong W.J., Li Y.S., Niu D.C., Ma Z., Gu J.L., Chen Y., Zhao W.R., Liu X.H., Liu C.S., Shi J.L. (2011). Facile Synthesis of Monodisperse Superparamagnetic Fe3O4 Core@hybrid@Au Shell Nanocomposite for Bimodal Imaging and Photothermal Therapy. Adv. Mater..

[55] 55. Kim J., Park S., Lee J.E., Jin S.M., Lee J.H., Lee I.S., Yang I., Kim J.S., Kim S.K., Cho M.H. (2006). Designed fabrication of multifunctional magnetic gold nanoshells and their application to magnetic resonance imaging and photothermal therapy. Angew. Chem..

[56] Salgueirino-Maceira V., Correa-Duarte M.A., Farle M., Lopez-Quintela A., Sieradzki K., Diaz R. (2006). Bifunctional gold-coated magnetic silica spheres. Chem. Mater..

[57] Lin C.-W., Chen J.-M., Lin Y.-J., Chao L.-W., Wei S.-Y., Wu C.-H., Jeng C.-C., Wang L.-M., Chen K.-L. (2019). Magneto-Optical Characteristics of Streptavidin-Coated Fe3O4@Au Core-Shell Nanoparticles for Potential Applications on Biomedical Assays. Sci. Rep..

[58] Ajinkya N., Yu X., Kaithal P., Luo H., Somani P., Ramakrishna S. (2020). Magnetic Iron Oxide Nanoparticle (IONP) Synthesis to Applications: Present and Future. Materials.

[59] Pham X.H., Hahm E., Kim H.M., Son B.S., Jo A., An J., Tran Thi T.A., Nguyen D.Q., Jun B. (2020). Silica-Coated Magnetic Iron Oxide Nanoparticles Grafted onto Graphene Oxide for Protein Isolation. Nanomaterials.

[60] Popova M., Koseva N., Trendafilova I., Lazarova H., Mitova V., Mihaly J., Momekova D., Konstantinov S., Koleva I.Z., Petkov P.S. (2021). Design of PEG-modified magnetic nanoporous silica based miltefosine delivery system: Experimental and theoretical approaches. Micropor. Mesopor. Mat..

[61] Popova M., Trendafilova I., Szegedi A., Momekova D., Mihaly J., Momekov G., Kiss L.F., Lazar K., Koseva N. (2018). Novel SO_3_H functionalized magnetic nanoporous silica/polymer nanocomposite as a carrier in a dual-drug delivery system for anticancer therapy. Micropor. Mesopor. Mat..

[62] Lee H.S., Kim E.H., Shao H., Kwak B.K. (2005). Synthesis of SPIO-chitosan microspheres for MRI-detectable embolotherapy. J. Magn. Magn. Mater..

[63] Cortés H., Hernández-Parra H., Bernal-Chávez S.A., Prado-Audelo M.L.D., Caballero-Florán I.H., Borbolla-Jiménez F.V., González-Torres M., Magaña J.J., Leyva-Gómez G. (2021). Non-Ionic Surfactants for Stabilization of Polymeric Nanoparticles for Biomedical Uses. Materials.

[64] Al-Jamal W.T., Kostarelos K. (2011). Liposomes: From a clinically established drug delivery system to a nanoparticle platform for theranostic nanomedicine. Acc. Chem. Res..

[65] Essa M.L., El-Kemary M.A., Ebrahem Saied E.M., Leporatti S., Nemany Hanafy N.A. (2020). Nano targeted Therapies Made of Lipids and Polymers have Promising Strategy for the Treatment of Lung Cancer. Materials.

[66] Su W., Wang H., Wang S., Liao Z., Kang S., Peng Y., Han L., Chang J. (2012). PEG/RGD-modified magnetic polymeric liposomes for controlled drug release and tumor cell targeting. Int. J. Pharm..

[67] Ai H., Flask C., Weinberg B., Shuai X.-T., Pagel M., Farrell D., Duerk J., Gao J. (2005). Magnetite-Loaded Polymeric Micelles as Ultrasensitive Magnetic-Resonance Probes. Adv. Mater..

[68] Wang J., Shah Z.H., Zhang S., Lu R. (2014). Silica-based nanocomposites via reverse microemulsions: Classifications, preparations, and applications. Nanoscale.

[69] Stephen Z.R., Kievit F.M., Zhang M. (2011). Magnetite Nanoparticles for Medical MR Imaging. Mater. Today.

[70] Frachini E., Petri D. (2019). Magneto-Responsive Hydrogels: Preparation, Characterization, Biotechnological and Environmental Applications. J. Braz. Chem. Soc..

[71] Wong H.L., Wu X.Y., Bendayan R. (2012). Nanotechnological advances for the delivery of CNS therapeutics. Adv. Drug Deliv. Rev..

[72] Omwoyo W.N., Ogutu B., Oloo F., Swai H., Kalombo L., Melariri P., Mahanga G.M., Gathirwa J.W. (2014). Preparation, characterization, and optimization of primaquine-loaded solid lipid nanoparticles. Int. J. Nanomed..

[73] Cavalli G., Banu S., Ranasinghe R.T., Broder G.R., Martins H.F., Neylon C., Morgan H., Bradley M., Roach P.L. (2007). Multistep synthesis on SU-8: Combining microfabrication and solid-phase chemistry on a single material. J. Comb. Chem..

[74] Marie R., Schmid S., Johansson A., Ejsing L., Nordström M., Häfliger D., Christensen C.B., Boisen A., Dufva M. (2006). Immobilisation of DNA to polymerised SU-8 photoresist. Biosens. Bioelectron..

[75] Tsakos M., Schaffert E., Clement L., Villadsen N., Poulsen T. (2015). Ester coupling reactions–an enduring challenge in the chemical synthesis of bioactive natural products. Nat. Prod. Rep..

[76] Azizi N., Kamrani P., Saadat M. (2016). A magnetic nanoparticle-catalyzed regioselective ring opening of epoxides by aromatic amines. Appl. Organomet. Chem..

[77] Gavrila H., Chiriac H., Ciureanu P., Ionita V., Yelon A. (2000). Magnetism Tehnic si Aplicat.

[78] Kolhatkar A.G., Jamison A.C., Litvinov D., Willson R.C., Lee T.R. (2013). Tuning the magnetic properties of nanoparticles. Int. J. Mol. Sci..

[79] Knobel M., Nunes W.C., Socolovsky L.M., Biasi E., Vargas J.M., Denardin J.C.J. (2008). Superparamagnetism and other magnetic features in granular materials: A review on ideal and real systems. Nanosci. Nanotech..

[80] Ferreira M., Sousa J., Pais A., Vitorino C. (2020). The Role of Magnetic Nanoparticles in Cancer Nanotheranostics. Materials.

[81] Pereira C., Pereira A.M., Fernandes C., Rocha M., Mendes R., Fernandez-Garcia M., Guedes A., Tavares P.B., Greneche J.-M., Araujo J.P. (2012). Superparamagnetic MFe_2_O_4_ (M = Fe, Co, Mn) nanoparticles: Tuning the particles size and magnetic properties through a novel one-step coprecipitation route. Chem. Mater..

[82] Guardia P., Labarta A., Batlle X. (2011). Tuning the size, the shape, and the magnetic properties of iron oxide nanoparticles. J. Phys. Chem. C.

[83] Caruntu D., Caruntu G., O’Connor C.J.J. (2007). Magnetic properties of variable-sized Fe_3_O_4_ nanoparticles synthesized from non-aqueous homogeneous solutions of polyols. Phys. D Appl. Phys..

[84] Noh S.H., Na W., Jang J.T., Lee J.H., Lee E.J., Moon S.H., Lim Y., Shin J.S., Cheon J. (2012). Nanoscale magnetism control via surface and exchange anisotropy for optimized ferrimagnetic hysteresis. Nano Lett..

[85] Zhen G., Muir B.W., Moffat B.A., Harbour P., Murray K.S., Moubaraki B., Suzuki K., Madsen I., Agron-Olshina N., Waddington L. (2011). Comparative study of magnetic behavior of spherical and cubic superparamagnetic iron oxide nanoparticles. J. Phys. Chem. C.

[86] Weissleder R., Stark D.D., Engelstad B.L., Bacon B.R., Compton C.C., White D.L., Jacobs P., Lewis J. (1989). Superparamagnetic iron oxide: Pharmacokinetics and toxicity. Am. J. Roentgenol..

[87] Wahajuddin A.S. (2012). Superparamagnetic iron oxide nanoparticles: Magnetic nanoplatforms as drug carriers. Int. J. Nanomed..

[88] Mukherjee S., Liang L., Veiseh O. (2020). Recent Advancements of magnetic nanomaterials in cancer therapy. Pharmaceutics.

[89] Silva F., Cabral Campello M.P., Paulo A. (2021). Radiolabeled Gold Nanoparticles for Imaging and Therapy of Cancer. Materials.

[90] Peer D., Karp J.M., Hong S., Farokhzad O.C., Margalit R., Langer R. (2007). Nanocarriers as an emerging platform for cancer therapy. Nat. Nanotechnol..

[91] Setyawati M.I., Tay C.Y., Leong D.T. (2014). The gap between endothelial cells: Key to the quick escape of nanomaterials?. Nanomedicine.

[92] Huang Y., Wang S., Zhang J.-Z., Wang H.-X., Wu L. (2021). Stealthy nanoparticles protecting endothelium barrier from leakiness by resisting the absorption of VE-cadherin. Nanoscale.

[93] Sehl O.C., Gevaert J.J., Melo K.P., Knier N.N., Foster P.J. (2020). A Perspective on Cell Tracking with Magnetic Particle Imaging. Tomography.

[94] Ardeshirpour Y., Chernomordik V., Hassan M., Zielinski R., Capala J., Gandjbakhche A. (2014). In vivo fluorescence lifetime imaging for monitoring the efficacy of the cancer treatment. Clin. Cancer Res..

[95] Wáng Y.X., Idée J.M. (2017). A comprehensive literatures update of clinical researches of superparamagnetic resonance iron oxide nanoparticles for magnetic resonance imaging. Quant. Imaging Med. Surg..

[96] Hong S.C., Lee J.H., Lee J., Kim H.Y., Park J.Y., Cho J., Lee J., Han D.W. (2011). Subtle cytotoxicity and genotoxicity differences in superparamagnetic iron oxide nanoparticles coated with various functional groups. Int. J. Nanomed..

[97] Basuki J.S., Duong H.T., Macmillan A., Erlich R.B., Esser L., Akerfeldt M.C., Whan R.M., Kavallaris M., Boyer C., Davis T.P. (2013). Using fluorescence lifetime imaging microscopy to monitor theranostic nanoparticle uptake and intracellular doxorubicin release. ACS Nano.

[98] Kaittanis C., Shaffer T.M., Ogirala A., Santra S., Perez J.M., Chiosis G., Li Y., Josephson L., Grimm J. (2014). Environment-responsive nanophores for therapy and treatment monitoring via molecular MRI quenching. Nat. Commun..

[99] Wang G., Serkova N.J., Groman E.V., Scheinman R.I., Simberg D. (2019). Feraheme (Ferumoxytol) is recognized by proinflammatory and anti-inflammatory macrophages via scavenger receptor type AI/II. Mol. Pharm..

[100] Chow A.Y. (2010). Cell cycle control by oncogenes and tumor supressors: Driving the transformation of normal cells into cancerous cells. Nat. Educ..

[101] Janko C., Ratschker T., Nguyen K., Zschiesche L., Tietze R., Lyer S., Alexiou C. (2019). Functionalized superparamagnetic iron oxide nanoparticles (SPIONs) as platform for the targeted multimodal tumor therapy. Front. Oncol..

[102] Khaledian M., Nourbakhsh M.S., Saber R., Hashemzadeh H., Darvishi M.H. (2020). Preparation and evaluation of Doxorubicin-oloaded PLA–PEG–FA copolymer containing superparamagnetic iron oxide nanoparticles (SPIONs) for cancer treatment: Combination therapy with hyperthermia and chemotherapy. Int. J. Nanomed..

[103] Raviraj V., Pham B.T.T., Kim B.J., Pham N.T.H., Kok L.F., Painter N., Delic N.C., Jones S.K., Hawkett B.S., Lyons J.G. (2021). Non-invasive transdermal delivery of chemotherapeutic molecules in vivo using superparamagnetic iron oxide nanoparticles. Cancer Nano.

[104] Zhang J., Stevens M.F., Bradshaw T.D. (2012). Temozolomide: Mechanism of action, repair and resistance. Curr. Mol. Pharmacol..

[105] Brooks L.J., Clements M.P., Burden J.J., Kocher D., Richards L., Devesa S.C., Zakka L., Woodberry M., Ellis M., Jaunmuktane Z. (2021). The white matter is a pro-differentiative niche for glioblastoma. Nat. Commun..

[106] Brandes A.A., Basso U., Reni M., Vastola F., Tosoni A., Cavallo L., Ferreri A.J., Panucci M.G., Monfardini S., Ermani M. (2004). First-line chemotherapy with cisplatin plus fractionated temozolomide in recurrent glioblastoma multiforme: A phase II study of the gruppo italiano cooperativo di neuro-oncologia. Clin. Oncol..

[107] Emamgholizadeh Minaei S., Khoei S., Khoee S., Karimi M.R. (2019). Tri-block copolymer nanoparticles modified with folic acid for temozolomide delivery in glioblastoma. Int. J. Biochem. Cell Biol..

[108] Caffery B., Lee J.S., Alexander-Bryant A.A. (2019). Vectors for Glioblastoma Gene Therapy: Viral & Non-Viral Delivery Strategies. Nanomaterials.

[109] Rego G.N.A., Nucci M.P., Mamani J.B., Oliveira F.A., Marti L.C., Filgueiras I.S., Ferreira J.M., Real C.C., Faria D.P., Espinha P.L. (2020). Therapeutic Efficiency of Multiple Applications of Magnetic Hyperthermia Technique in Glioblastoma Using Aminosilane Coated Iron Oxide Nanoparticles: In Vitro and In Vivo Study. Int. J. Mol. Sci..

[110] Zhao M., van Straten D., Broekman M., Préat V., Schiffelers R.M. (2020). Nanocarrier-based drug combination therapy for glioblastoma. Theranostics.

[111] Zargar H., Aning J., Ischia J., So A., Black P. (2014). Optimizing intravesical mitomycin C therapy in non-muscle-invasive bladder cancer. Nat. Rev. Urol..

[112] Eldin O.S., Fouda A.M., Youssef A.R., Hamilton P., Maxwell P., Williamson K.E. (2018). Reduction of mitomycin C resistance in human bladder cancer T24 cells by knocking-down ras oncogene. Cancer Drug Resist..

[113] Scotté F., Ratta R., Beuzeboc P. (2019). Side effects of immunotherapy: A constant challenge for oncologists. Curr. Opin. Oncol..

[114] Baselga J., Albanell J. (2001). Mechanism of action of anti-HER2 monoclonal antibodies. Ann. Oncol..

[115] Filin I.Y., Solovyeva V.V., Kitaeva K.V., Rutland C.S., Rizvanov A.A. (2020). Current Trends in Cancer Immunotherapy. Biomedicines.

[116] Mahmoudi M., Sant S., Wang B., Laurent S., Sen T. (2011). Superparamagnetic iron oxide nanoparticles (SPIONs): Development, surface modification and applications in chemotherapy. Adv. Drug Deliv. Rev..

[117] Crețu B.E., Dodi G., Shavandi A., Gardikiotis I., Șerban I.L., Balan V. (2021). Imaging Constructs: The Rise of Iron Oxide Nanoparticles. Molecules.

[118] ThermoFisher SCIENTIFIC. https://www.thermofisher.com/ro/en/home/life-science/cell-analysis/cell-analysis-learning-center/immunology-at-work/macrophage-cell-overview.html.

[119] ThermoFisher SCIENTIFIC. https://www.thermofisher.com/ro/en/home/life-science/cell-analysis/cell-analysis-learning-center/immunology-at-work/dendritic-cell-overview.html.

[120] Gires O., Pan M., Schinke H., Canis M., Baeuerle P.A. (2020). Expression and function of epithelial cell adhesion molecule EpCAM: Where are we after 40 years?. Cancer Metastasis Rev..

[121] Shapiro C.L., Recht A. (2001). Side effects of adjuvant treatment of breast cancer. N. Engl. J. Med..

[122] Ceeraz S., Nowak E.C., Burns C.M., Noelle R.J. (2014). Immune checkpoint receptors in regulating immune reactivity in rheumatic disease. Arthritis Res. Ther..

[123] Freeman G.J., Long A.J., Iwai Y., Bourque K., Chernova T., Nishimura H., Fitz L.J., Malenkovich N., Okazaki T., Byrne M.C. (2000). Engagement of the PD-1 immunoinhibitory receptor by a novel B7 family member leads to negative regulation of lymphocyte activation. J. Exp. Med..

[124] Leach D.R., Krummel M.F., Allison J.P. (1996). Enhancement of antitumor immunity by CTLA-4 blockade. Science.

[125] Schmid D., Park C.G., Hartl C.A., Subedi N., Cartwright A.N., Puerto R.B., Zheng Y., Maiarana J., Freeman G.J., Wucherpfennig K.W. (2017). T cell-targeting nanoparticles focus delivery of immunotherapy to improve antitumor immunity. Nat. Commun..

[126] Mühlberger M., Janko C., Unterweger H., Schreiber E., Band J., Lehmann C., Dudziak D., Lee G., Alexiou C., Tietze R. (2019). Functionalization of T lymphocytes for magnetically controlled immune therapy: Selection of suitable superparamagnetic iron oxide nanoparticles. J. Magn. Magn. Mater..

[127] Shaik A.P., Shaik A.S., Al Majwal A., Al Faraj A. (2017). Blocking Interleukin-4 Receptor α Using Polyethylene Glycol Functionalized Superparamagnetic Iron Oxide Nanocarriers to Inhibit Breast Cancer Cell Proliferation. Cancer Res. Treat..

[128] Lunov O., Uzhytchak M., Smolková B., Lunova M., Jirsa M., Dempsey N.M., Dias A.L., Bonfim M., Hof M., Jurkiewicz P. (2019). Remote Actuation of Apoptosis in Liver Cancer Cells via Magneto-Mechanical Modulation of Iron Oxide Nanoparticles. Cancers.

[129] Hao H., Ma Q., He F., Yao P. (2014). Doxorubicin and Fe3O4 Loaded Albumin Nanoparticles with Folic Acid Modified Dextran Surface for Tumor Diagnosis and Therapy. J. Mater. Chem. B.

[130] Comes Franchini M., Baldi G., Bonacchi D., Gentili D., Giudetti G., Lascialfari A., Corti M., Marmorato P., Ponti J., Micotti E. (2010). Bovine Serum Albumin-Based Magnetic Nanocarrier for MRI Diagnosis and Hyperthermic Therapy: A Potential Theranostic Approach against Cancer. Small.

[131] Yu S.-M., Gonzalez-Moragas L., Milla M., Kolovou A., Santarella-Mellwig R., Schwab Y., Laromaine A., Roig A. (2016). Bio-Identity and Fate of Albumin-Coated SPIONs Evaluated in Cells and by the C. elegans Model. Acta Biomater..

[132] Vidawati S., Barbosa S., Taboada P., Villar E., Topete A., Mosquera V. (2018). Study of Human Serum Albumin-SPIONs Loaded PLGA Nanoparticles for Protein Delivery. Adv. Biol. Chem..

[133] Subbiahdoss G., Sharifi S., Grijpma D.W., Laurent S., Van Der Mei H.C., Mahmoudi M., Busscher H.J. (2012). Magnetic Targeting of Surface-Modified Superparamagnetic Iron Oxide Nanoparticles Yields Antibacterial Efficacy against Biofilms of Gentamicin-Resistant Staphylococci. Acta Biomater..

[134] Dadfar S.M., Roemhild K., Drude N.I., von Stillfried S., Knüchel R., Kiessling F., Lammers T. (2019). Iron Oxide Nanoparticles: Diagnostic, Therapeutic and Theranostic Applications. Adv. Drug Deliv. Rev..

[135] Fang C., Wang K., Stephen Z.R., Mu Q., Kievit F.M., Chiu D.T., Press O.W., Zhang M. (2015). Temozolomide Nanoparticles for Targeted Glioblastoma Therapy. ACS Appl. Mater. Interfaces.

[136] Hu S.H., Liao B.J., Chiang C.S., Chen P.J., Chen I.W., Chen S.Y. (2012). Core-Shell Nanocapsules Stabilized by Single-Component Polymer and Nanoparticles for Magneto-Chemotherapy/Hyperthermia with Multiple Drugs. Adv. Mater..

[137] Sun C., Du K., Fang C., Bhattarai N., Veiseh O., Kievit F., Stephen Z., Lee D., Ellenbogen R.G., Ratner B. (2010). PEG Mediated Synthesis of Highly Dispersive Multifunctional Superparamagnetic Nanoparticles: Their Physicochemical Properties and Function in Vivo. ACS Nano.

[138] Alupei L., Peptu C.A., Lungan A.M., Desbrieres J., Chiscan O., Radji S., Popa M. (2016). New Hybrid Magnetic Nanoparticles Based on Chitosan-Maltose Derivative for Antitumor Drug Delivery. Int. J. Biol. Macromol..

[139] Shakeri-Zadeh A., Khoee S., Shiran M.B., Sharifi A.M., Khoei S. (2015). Synergistic Effects of Magnetic Drug Targeting Using a Newly Developed Nanocapsule and Tumor Irradiation by Ultrasound on CT26 Tumors in BALB/c Mice. J. Mater. Chem. B.

[140] Shakeri-Zadeh A., Shiran M.B., Khoee S., Sharifi A.M., Ghaznavi H., Khoei S. (2014). A New Magnetic Nanocapsule Containing 5- Fluorouracil: In Vivo Drug Release, Anti-Tumor, and pro-Apoptotic Effects on CT26 Cells Allograft Model. J. Biomater. Appl..

[141] Russell E., Dunne V., Russell B., Mohamud H., Ghita M., McMahon S.J., Butterworth K.T., Schettino G., McGarry C.K., Prise K.M. (2021). Impact of superparamagnetic iron oxide nanoparticles on in vitro and in vivo radiosensitisation of cancer cells. Radiat. Oncol..

[142] Dulińska-Litewka J., Łazarczyk A., Hałubiec P., Szafrański O., Karnas K., Karewicz A. (2019). Superparamagnetic Iron Oxide Nanoparticles-Current and Prospective Medical Applications. Materials.

[143] Lee S.H., Kim B.H., Na H.B., Hyeon T. (2014). Paramagnetic inorganic nanoparticles as T1 MRI contrast agents. Wiley Interdiscip. Rev.: Nanomed. Nanobiotechnol..

[144] Hu P., Fu Z., Liu G., Tan H., Xiao J., Shi H., Cheng D. (2019). Gadolinium-Based Nanoparticles for Theranostic MRI-Guided Radiosensitization in Hepatocellular Carcinoma. Front. Bioeng. Biotechnol..

[145] Klein S., Sommer A., Distel L.V., Neuhuber W., Kryschi C. (2012). Superparamagnetic iron oxide nanoparticles as radiosensitizer via enhanced reactive oxygen species formation. Biochem. Biophys. Res. Commun..

[146] Kirakli E., Takan G., Hoca S., Muftuler F.Z. (2018). Superparamagnetic iron oxide nanoparticle (SPION) mediated in vitro radiosensitization at megavoltage radiation energies. J. Radioanal. Nucl. Chem..

[147] Abdul Rashid R., Zainal Abidin S., Khairil Anuar M.A., Tominaga T., Akasaka H., Sasaki R., Kie K., Razak K.A., Pham B.T.T., Hawkett B.S. (2019). Radiosensitization effects and ROS generation by high Z metallic nanoparticles on human colon carcinoma cell (HCT116) irradiated under 150 MeV proton beam. OpenNano.

[148] Choi G.H., Seo S.J., Kim K.H., Kim H.T., Park S.H., Lim J.H., Kim J.K. (2012). Photon activated therapy (PAT) using monochromatic synchrotron X-rays and iron oxide nanoparticles in a mouse tumor model: Feasibility study of PAT for the treatment of superficial malignancy. Radiat. Oncol..

[149] Mesbahi A. (2010). A review on gold nanoparticles radiosensitization effect in radiation therapy of cancer. Rep. Pract. Oncol. Radiother..

[150] Zhang X., Liu Z., Lou Z., Chen F., Chang S., Miao Y., Zhou Z., Hu X., Feng J., Ding Q. (2018). Radiosensitivity enhancement of Fe_3_O_4_@Ag nanoparticles on human glioblastoma cells. Artif. Cells Nanomed. Biotechnol..

[151] Xu R., Ma J., Sun X., Chen Z., Jiang X., Guo Z., Huang L., Li Y., Wang M., Wang C. (2009). Ag nanoparticles sensitize IR-induced killing of cancer cells. Cell Res..

[152] Liu P., Huang Z., Chen Z., Xu R., Wu H., Zang F., Wang C., Gu N. (2013). Silver nanoparticles: A novel radiation sensitizer for glioma?. Nanoscale.

[153] Wu H., Lin J., Liu P., Huang Z., Zhao P., Jin H., Ma J., Wen L., Gu N. (2016). Reactive oxygen species acts as executor in radiation enhancement and autophagy inducing by AgNPs. Biomaterials.

[154] Wu H., Lin J., Liu P., Huang Z., Zhao P., Jin H., Wang C., Wen L., Gu N. (2015). Is the autophagy a friend or foe in the silver nanoparticles associated radiotherapy for glioma?. Biomaterials.

[155] Liu P., Jin H., Guo Z., Ma J., Zhao J., Li D., Wu H., Gu N. (2016). Silver nanoparticles outperform gold nanoparticles in radiosensitizing U251 cells in vitro and in an intracranial mouse model of glioma. Int. J. Nanomed..

[156] Eriksson D., Stigbrand T. (2010). Radiation-induced cell death mechanisms. Tumour. Biol..

[157] Zhao D., Sun X., Tong J., Ma J., Bu X., Xu R., Fan R. (2012). A novel multifunctional nanocomposite C225-conjugated Fe3O4/Ag enhances the sensitivity of nasopharyngeal carcinoma cells to radiotherapy. Acta Biochim. Biophys. Sin..

[158] Datta N.R., Ordóñez S.G., Gaipl U.S., Paulides M.M., Crezee H., Gellermann J., Marder D., Puric E., Bodis S. (2015). Local hyperthermia combined with radiotherapy and-/or chemotherapy: Recent advances and promises for the future. Cancer Treat. Rev..

[159] Bañobre-López M., Teijeiro A., Rivas J. (2013). Magnetic nanoparticle-based hyperthermia for cancer treatment. Rep. Pract. Oncol. Radiother..

[160] Gilchrist R.K., Medal R., Shorey W.D., Hanselman R.C., Parrott J.C., Taylor C.B. (1957). Selective inductive heating of lymph nodes. Ann. Surg..

[161] Blanco-Andujar C., Teran F.J., Ortega D., Mahmoudi M., Laurent S. (2018). Current outlook and perspectives on nanoparticle-mediated magnetic hyperthermia. Iron Oxide Nanoparticles for Biomedical Applications.

[162] Kumar C.S., Mohammad F. (2011). Magnetic nanomaterials for hyperthermia-based therapy and controlled drug delivery. Adv. Drug Deliv. Rev..

[163] Shubayev V.I., Pisanic T.R., Jin S. (2009). Magnetic nanoparticles for theragnostics. Adv. Drug Deliv. Rev..

[164] Yoo D., Lee J.H., Shin T.H., Cheon J. (2011). Theranostic magnetic nanoparticles. Acc. Chem. Res..

[165] Ho D., Sun X., Sun S. (2011). Monodisperse magnetic nanoparticles for theranostic applications. Acc. Chem. Res..

[166] Espinosa A., Curcio A., Cabana S., Radtke G., Bugnet M., Kolosnjaj-Tabi J., Péchoux C., Alvarez-Lorenzo C., Botton G.A., Silva A.K.A. (2018). Intracellular Biodegradation of Ag Nanoparticles, Storage in Ferritin, and Protection by a Au Shell for Enhanced Photothermal Therapy. ACS Nano.

[167] Hildebrandt B., Wust P., Ahlers O., Dieing A., Sreenivasa G., Kerner T., Felix R., Riess H. (2002). The cellular and molecular basis of hyperthermia. Crit. Rev. Oncol. Hematol..

[168] Ong Y.S., Bañobre-López M., Costa Lima S.A., Reis S. (2020). A multifunctional nanomedicine platform for co-delivery of methotrexate and mild hyperthermia towards breast cancer therapy. Mater. Sci. Eng. C Mater. Biol. Appl..

[169] Datta N.R., Krishnan S., Speiser D.E., Neufeld E., Kuster N., Bodis S., Hofmann H. (2016). Magnetic nanoparticle-induced hyperthermia with appropriate payloads: Paul Ehrlich’s "magic (nano)bullet" for cancer theranostics?. Cancer Treat. Rev..

[170] Jordan A., Scholz R., Wust P., Fähling H., Felix R. (1999). Magnetic fluid hyperthermia (MFH): Cancer treatment with AC magnetic field induced excitation of biocompatible superparamagnetic nanoparticles. J. Magn. Magn. Mater..

[171] Rosenweig R.E. (2002). Heating magnetic fluid with alternating magnetic field. J. Magn. Magn. Mater..

[172] Lee J.H., Jang J.T., Choi J.S., Moon S.H., Noh S.H., Kim J.W., Kim J.G., Kim I.S., Park K.I., Cheon J. (2011). Exchange-coupled magnetic nanoparticles for efficient heat induction. Nat. Nanotechnol..

[173] Liu X.L., Ng C.T., Chandrasekharan P., Yang H.T., Zhao L.Y., Peng E., Lv Y.B., Xiao W., Fang J., Yi J.B. (2016). Synthesis of Ferromagnetic Fe_0.6_ Mn_0.4_ O Nanoflowers as a New Class of Magnetic Theranostic Platform for In Vivo T1 -T2 Dual-Mode Magnetic Resonance Imaging and Magnetic Hyperthermia Therapy. Adv. Healthc. Mater..

[174] Jang J.T., Nah H., Lee J.H., Moon S.H., Kim M.G., Cheon J. (2009). Critical enhancements of MRI contrast and hyperthermic effects by dopant-controlled magnetic nanoparticles. Angew. Chem. Int. Ed..

[175] Chen R., Christiansen M.G., Anikeeva P. (2013). Maximizing hysteretic losses in magnetic ferrite nanoparticles via model-driven synthesis and materials optimization. ACS Nano.

[176] Jang J.T., Lee J., Seon J., Ju E., Kim M., Kim Y.I., Kim M.G., Takemura Y., Arbab A.S., Kang K.W. (2018). Giant Magnetic Heat Induction of Magnesium-Doped γ-Fe_2_ O_3_ Superparamagnetic Nanoparticles for Completely Killing Tumors. Adv. Mater..

[177] Liu X., Peng M., Li G., Miao Y., Luo H., Jing G., He Y., Zhang C., Zhang F., Fan H. (2019). Ultrasonication-Triggered Ubiquitous Assembly of Magnetic Janus Amphiphilic Nanoparticles in Cancer Theranostic Applications. Nano Lett..

[178] Du Y., Liu X., Liang Q., Liang X.J., Tian J. (2019). Optimization and Design of Magnetic Ferrite Nanoparticles with Uniform Tumor Distribution for Highly Sensitive MRI/MPI Performance and Improved Magnetic Hyperthermia Therapy. Nano Lett..

[179] Sugumaran P.J., Liu X.L., Herng T.S., Peng E., Ding J. (2019). GO-Functionalized Large Magnetic Iron Oxide Nanoparticles with Enhanced Colloidal Stability and Hyperthermia Performance. ACS Appl. Mater. Interfaces.

[180] Liu X.L., Yang Y., Ng C.T., Zhao L.Y., Zhang Y., Bay B.H., Fan H.M., Ding J. (2015). Magnetic vortex nanorings: A new class of hyperthermia agent for highly efficient in vivo regression of tumors. Adv. Mater..

[181] Clerc P., Jeanjean P., Hallali N., Gougeon M., Pipy B., Carrey J., Fourmy D., Gigoux V. (2018). Targeted Magnetic Intra-Lysosomal Hyperthermia produces lysosomal reactive oxygen species and causes Caspase-1 dependent cell death. J. Control. Release.

[182] Domenech M., Marrero-Berrios I., Torres-Lugo M., Rinaldi C. (2013). Lysosomal membrane permeabilization by targeted magnetic nanoparticles in alternating magnetic fields. ACS Nano.

[183] de la Fuente J.M., Grazu V. (2012). Nanobiotechnology, Inorganic Nanoparticles vs Organic Nanoparticles.

[184] Kozissnik B., Bohorquez A.C., Dobson J., Rinaldi C. (2013). Magnetic fluid hyperthermia: Advances, challenges, and opportunity. Int. J. Hyperth..

[185] Andreu I., Natividad E. (2013). Accuracy of available methods for quantifying the heat power generation of nanoparticles for magnetic hyperthermia. Int. J. Hyperth..

[186] Riedinger A., Guardia P., Curcio A., Garcia M.A., Cingolani R., Manna L., Pellegrino T. (2013). Subnanometer local temperature probing and remotely controlled drug release based on azo-functionalized iron oxide nanoparticles. Nano Lett..

[187] Albarqi H.A., Wong L.H., Schumann C., Sabei F.Y., Korzun T., Li X., Hansen M.N., Dhagat P., Moses A.S., Taratula O. (2019). Biocompatible Nanoclusters with High Heating Efficiency for Systemically Delivered Magnetic Hyperthermia. ACS Nano.

[188] Ito A., Shinkai M., Honda H., Yoshikawa K., Saga S., Wakabayashi T., Yoshida J., Kobayashi T. (2003). Heat shock protein 70 expression induces antitumor immunity during intracellular hyperthermia using magnetite nanoparticles. Cancer Immunol. Immunother..

[189] Yanase M., Shinkai M., Honda H., Wakabayashi T., Yoshida J., Kobayashi T. (1997). Intracellular hyperthermia for cancer using magnetite cationic liposomes: Ex vivo study. Jpn. J. Cancer Res..

[190] Yanase M., Shinkai M., Honda H., Wakabayashi T., Yoshida J., Kobayashi T. (1998). Antitumor immunity induction by intracellular hyperthermia using magnetite cationic liposomes. Jpn. J. Cancer Res..

[191] Rabias I., Tsitrouli D., Karakosta E., Kehagias T., Diamantopoulos G., Fardis M., Stamopoulos D., Maris T.G., Falaras P., Zouridakis N. (2010). Rapid magnetic heating treatment by highly charged maghemite nanoparticles on Wistar rats exocranial glioma tumors atmicroliter volume. Biomicrofluidics.

[192] Ito A., Shinkai M., Honda H., Kobayashi T. (2001). Heat-inducible TNF-alpha gene therapy combined with hyperthermia using magnetic nanoparticles as a novel tumor-targeted therapy. Cancer Gene Ther..

[193] van Landeghem F.K., Maier-Hauff K., Jordan A., Homann K.T., Gneveckow U., Scholz R., Thiesen B., Bruck W., von Deimling A. (2009). Post-mortem studies in glioblastoma patients treated with thermotherapy using magnetic nanoparticles. Biomaterials.

[194] Gupta R., Sharma D. (2019). Biofunctionalization of magnetite nanoparticles with stevioside: Effect on the size and thermal behaviour for use in hyperthermia applications. Int. J. Hyperth..

[195] Maier-Hauff K., Rothe R., Scholz R., Gneveckow U., Wust P., Thiesen B., Feussner A., von Deimling A., Waldoefner N., Felix R. (2007). Intracranial thermotherapy using magnetic nanoparticles combined with external beam radiotherapy: Results of a feasibility study on patients with glioblastoma multiforme. J. Neuro -Oncol..

[196] Maier-Hauff K., Ulrich F., Nestler D., Nieho H., Wust P., Thiesen B., Orawa H., Budach V., Jordan A. (2011). Efficacy and safety of intratumoral thermotherapy using magnetic iron-oxide nanoparticles combined with external beam radiotherapy on patients with recurrent glioblastoma multiforme. J. Neuro -Oncol..

[197] Wismeth C., Dudel C., Pascher C., Ramm P., Pietsch T., Hirschmann B., Reinert C., Proescholdt M., Rummele P., Schuierer G. (2010). Transcranial electro-hyperthermia combined with alkylating chemotherapy in patients with relapsed high-grade gliomas: Phase I clinical results. J. Neuro -Oncol..

[198] Kekalo K., Baker I., Meyers R., Shyong J. (2015). Magnetic Nanoparticles with High Specific Absorption Rate at Low Alternating Magnetic Field. Nano Life.

[199] Hanini A., Lartigue L., Gavard J., Schmitt A., Kacem K., Wilhelm C., Gazeau F., Chau F., Ammar S. (2016). Thermosensitivity profile of malignant glioma U87-MG cells and human endothelial cells following γ-Fe_2_O_3_ NPs internalization and magnetic field application. RSC Adv..

[200] Hanini A., Lartigue L., Gavard J., Kacem K., Wilhelm C., Gazeau F., Chau F., Ammar S. (2016). Zinc substituted ferrite nanoparticles with Zn_0.9_Fe_2.1_O_4_ formula used as heating agents for in vitro hyperthermia assay on glioma cells. J. Magn. Magn. Mater..

[201] Shevtsov M.A., Multhoff G. (2016). Recent developments of magnetic nanoparticles for theranostics of brain tumor. Curr. Drug Metab..

[202] Shinkai M., Yanase M., Honda H., Wakabayashi T., Yoshida J., Kobayashi T. (1996). Intracellular Hyperthermia for Cancer Using Magnetite Cationic Liposomes: In vitro Study. Jpn. J. Cancer Res..

[203] Meenach S.A., Hilt J.Z., Anderson K.W. (2010). Poly(ethylene glycol)-based magnetic hydrogel nanocomposites for hyperthermia cancer therapy. Acta Biomater..

[204] Altanerova U., Babincova M., Babinec P., Benejova K., Jakubechova J., Altanerova V., Zduriencikova M., Repiska V., Altaner C. (2017). Human mesenchymal stem cell-derived iron oxide exosomes allow targeted ablation of tumor cells via magnetic hyperthermia. Int. J. Nanomed..

[205] Mannucci S., Tambalo S., Conti G., Ghin L., Milanese A., Carboncino A., Nicolato E., Marinozzi M.R., Benati D., Bassi R. (2018). Magnetosomes Extracted from Magnetospirillum gryphiswaldense as Theranostic Agents in an Experimental Model of Glioblastoma. Contrast Media Mol. Imaging.

[206] Orlando T., Mannucci S., Fantechi E., Conti G., Tambalo S., Busato A., Innocenti C., Ghin L., Bassi R., Arosio P. (2016). Characterization of magnetic nanoparticles from Magnetospirillum Gryphiswaldense as potential theranostics tools. Contrast Media Mol. Imaging.

[207] Grapa C.M., Mocan L., Crisan D., Florea M., Mocan T. (2021). Biomarkers in Pancreatic Cancer as Analytic Targets for Nanomediated Imaging and Therapy. Materials.

[208] Cheng R., Feng F., Meng F., Deng C., Feijen J., Zhong Z. (2011). Glutathione-responsive nano-vehicles as a promising platform for targeted intracellular drug and gene delivery. J. Control. Release.

[209] Kievit F.M., Stephen Z.R., Wang K., Dayringer C.J., Sham J.G., Ellenbogen R.G., Silber J.R., Zhang M. (2015). Nanoparticle mediated silencing of DNA repair sensitizes pediatric brain tumor cells to γ-irradiation. Mol. Oncol..

[210] Revia R.A., Zhang M. (2016). Magnetite nanoparticles for cancer diagnosis, treatment, and treatment monitoring: Recent advances. Mater Today.

[211] Huber D.L. (2005). Synthesis, properties, and applications of iron nanoparticles. Small.

[212] Stockwell B.R., Friedmann Angeli J.P., Bayir H., Bush A.I., Conrad M., Dixon S.J., Fulda S., Gascón S., Hatzios S.K., Kagan V.E. (2017). Ferroptosis: A Regulated Cell Death Nexus Linking Metabolism, Redox Biology, and Disease. Cell.

[213] Chen X., Kang R., Kroemer G., Tang D. (2021). Broadening horizons: The role of ferroptosis in cancer. Nat. Rev. Clin. Oncol..

[214] Battaglia A.M., Chirillo R., Aversa I., Sacco A., Costanzo F., Biamonte F. (2020). Ferroptosis and Cancer: Mitochondria Meet the "Iron Maiden" Cell Death. Cells.

[215] Dixon S.J., Stockwell B.R. (2019). The hallmarks of ferroptosis. Annu. Rev. Cancer Biol..

[216] Lu B., Chen X.B., Ying M.D., He Q.J., Cao J., Yang B. (2018). The Role of Ferroptosis in Cancer Development and Treatment Response. Front. Pharmacol..

[217] Dixon S.J., Lemberg K.M., Lamprecht M.R., Skouta R., Zaitsev E.M., Gleason C.E., Patel D.N., Bauer A.J., Cantley A.M., Yang W.S. (2012). Ferroptosis: An iron-dependent form of nonapoptotic cell death. Cell.

[218] DeHart D.N., Fang D., Heslop K., Li L., Lemasters J.J., Maldonado E.N. (2018). Opening of voltage dependent anion channels promotes reactive oxygen species generation, mitochondrial dysfunction and cell death in cancer cells. Biochem. Pharmacol..

[219] Agmon E., Solon J., Bassereau P., Stockwell B.R. (2018). Modeling the effects of lipid peroxidation during ferroptosis on membrane properties. Sci. Rep..

[220] Su L.-J., Zhang J.-H., Gomez H., Murugan R., Hong X., Xu D., Jiang F., Peng Z.-Y. (2019). Review Article reactive oxygen species-induced lipid peroxidation in apoptosis, autophagy, and ferroptosis. Oxidative Med. Cell. Longev..

[221] Xie Y., Hou W., Song X., Yu Y., Huang J., Sun X., Kang R., Tang D. (2016). Ferroptosis: Process and function. Cell Death Differ..

[222] Mou Y., Wang J., Wu J., He D., Zhang C., Duan C., Li B. (2019). Ferroptosis, a new form of cell death: Opportunities and challenges in cancer. J. Hematol. Oncol..

[223] Shen Z., Song J., Yung B.C., Zhou Z., Wu A., Chen X. (2018). Emerging Strategies of Cancer Therapy Based on Ferroptosis. Adv. Mater..

[224] Ahmad T., Sarwar R., Iqbal A., Bashir U., Farooq U., Halim S.A., Khan A., Harassi A. (2020). Recent advances in combinatorial cancer therapy via multifunctionalized gold nanoparticles. Nanomedicine.

[225] Patil R.M., Thorat N.D., Shete P.B., Bedge P.A., Gavde S., Joshi M.G., Tofail S.A.M., Bohara R.A. (2018). Comprehensive cytotoxicity studies of superparamagnetic iron oxide nanoparticles. Biochem. Biophys. Rep..

[226] Hassannia B., Vandenabeele P., Vanden Berghe T. (2019). Targeting Ferroptosis to Iron Out Cancer. Cancer Cell.

[227] Farzin A., Etesami S.A., Quint J., Memic A., Tamayol A. (2020). Magnetic Nanoparticles in Cancer Therapy and Diagnosis. Adv. Healthc. Mater..

[228] Wu K., Su D., Liu J., Saha R., Wang J.P. (2019). Magnetic nanoparticles in nanomedicine: A review of recent advances. Nanotechnology.

[229] Cavalu S., Fritea L., Brocks M., Barbaro K., Murvai G., Costea T.O., Antoniac I., Verona C., Romani M., Latini A. (2020). Novel hybrid composites based on PVA/SeTiO_2_ nanoparticles and natural hydroxyapatite for orthopedic applications: Correlations between structural, morphological and biocompatibility properties. Materials.

[230] Laptoiu D., Marinescu R., Balan C., Antoniac I. (2015). Rheologic properties of some current hyaluronic acid products for viscosupplimentation-new trends for amelioration. Mater. Plast..

[231] Petreus T., Stoica B.A., Petreus O., Goriuc A., Cotrutz C.E., Antoniac I.V., Barbu-Tudoran L. (2014). Preparation and cytocompatibilityevaluation for hydrosolublephosphorous acid-derivatizedcellulose as tissue engineering scaffold material. J. Mater. Sci. Mater. Med..

[232] Cojocaru F.D., Balan V., Popa M.I., Lobiuc A., Antoniac A., Antoniac I.V., Verestiuc L. (2019). Biopolymers - Calcium phosphates composites with inclusions of magnetic nanoparticles for bone tissue engineering. Int. J. Biol. Macromol..

[233] Guazzo R., Gardin C., Bellin G., Sbricoli L., Ferroni L., Ludovichetti F.S., Piattelli A., Antoniac I., Bressan E., Zavan B. (2018). Graphene-based nanomaterials for tissue engineering in the dental field. Nanomaterials.

[234] Sarosi C., Biris A.R., Antoniac A., Boboia S., Alb C., Antoniac I., Moldovan M. (2016). The nanofiller effect on properties of experimental graphene dental nanocomposites. J. Adhes. Sci. Technol..

[235] Sinescu C., Marsavina L., Negrutiu M.L., Rusu L.C., Ardelean L., Rominu M., Antoniac I., Topala F.I., Podoleanu A. (2012). New metallic nanoparticles modified adhesive used for time domain optical coherence tomography evaluation of class II direct composite restoration. Revista Chimie.

[236] Cavalu S., Kamel E., Laslo V., Fritea L., Costea T., Antoniac I.V., Vasile E., Antoniac A., Semenescu A., Mohan A. (2017). Eco-friendly, facile and rapid way for synthesis of selenium nanoparticles production, structural and morphological characterization. Revista Chimie.

[237] Cavalu S., Antoniac I.V., Mohan A., Bodog F., Doicin C., Mates I., Ulmeanu M., Murzac R., Semenescu A. (2020). Nanoparticles and Nanostructured Surface Fabrication for Innovative Cranial and Maxillofacial Surgery. Materials.

[238] Cavalu S., Antoniac I.V., Fritea L., Mates I.M., Milea C., Laslo V., Vicas S., Mohan A. (2018). Surface modifications of the titanium mesh for cranioplasty using selenium nanoparticles coating. J. Adhes. Sci. Technol..

[239] Szegedi A., Trendafilova I., Mihaly J., Lazar K., Nemeth P., Momekov G., Momekova D., Marinov L., Nikolova I., Popova M. (2020). New insight on prednisolone polymorphs in mesoporous silica/maghemite nanocomposites. J. Drug Deliv. Sci. Technol..

[240] Radeloff K., Ramos Tirado M., Haddad D., Breuer K., Müller J., Hochmuth S., Hackenberg S., Scherzad A., Kleinsasser N., Radeloff A. (2021). Superparamagnetic Iron Oxide Particles (VSOPs) Show Genotoxic Effects but No Functional Impact on Human Adipose Tissue-Derived Stromal Cells (ASCs). Materials.

[241] Ruiz-Pulido G., Medina D.I., Barani M., Rahdar A., Sargazi G., Baino F., Pandey S. (2021). Nanomaterials for the Diagnosis and Treatment of Head and Neck Cancers: A Review. Materials.

